# HIGD1A Alleviates Oxidative Stress Related Ovarian Hypofunction by Enhancing Granulosa Cell Functions via NF‐κB/SOD2 Signaling Pathway

**DOI:** 10.1002/advs.202503828

**Published:** 2025-08-13

**Authors:** Huiying Li, Siying Cai, Hongbei Mu, Tianli Chang, Xiaofei Wang, Tianyu Tang, Tengyun Dong, Yu Zhang, Ling Zhang, Huaibiao Li, Liquan Zhou, Wenpei Xiang

**Affiliations:** ^1^ Institute of Reproductive Health Tongji Medical College Huazhong University of Science and Technology Wuhan Hubei 430030 China; ^2^ Department of Obstetrics and Gynecology The First College of Clinical Medical Science Yichang Central People's Hospital, Three Gorges University 183 Yiling Street Yichang Hubei 443003 China; ^3^ Reproductive Medicine Center Xiangya Hospital, Central South University Changsha Hunan 410008 China; ^4^ Wuhan Optics Valley Vcanbio Cell & Gene Technology Co., Ltd. Wuhan Hubei 430200 China; ^5^ Hubei Engineering Research Center for Human Stem Cell Preparation Application and Resource Preservation Wuhan Hubei 430014 China

**Keywords:** cellular senescence, diminished ovarian reserve, granulosa cells, HIGD1A, oxidative stress, reactive oxygen species

## Abstract

Diminished ovarian reserve (DOR) is a physiological or pathological condition that progresses in an age‐dependent manner, which is characterized by impaired ovarian follicle quality, decreased anti‐Müllerian hormone levels, elevated follicle‐stimulating hormone levels, and reduced antral follicle counts. Oxidative stress (OS) is one of the culprits of DOR. By imposing OS damage on various kinds of ovarian cells including granulosa cells, OS can result in ovarian hypofunction and eventually lead to female infertility. However, the underlying mechanisms have not been fully elucidated yet. In this study, HIGD1A, a mitochondrial inner membrane component, is found to be downregulated in granulosa cells upon OS exposure. By systematically studying the role of *HIGD1A* in regulating granulosa cell and ovarian functions as well as its corresponding mechanisms, a novel regulatory mechanism underlying OS‐related female infertility is revealed, and provided a potential molecular target for anti‐OS therapies.

## Graphical abstract

HIGD1A maintains redox homeostasis in granulosa cells by modulating SOD2‐mediated dismutation of superoxide into H_2_O_2_, which is cleared by CAT and GPX enzymes. However, reduced HIGD1A activates the NF‐κB pathway and upregulates SOD2, leading to intracellular H_2_O_2_ accumulation due to impaired clearance. Concurrently, low HIGD1A suppresses NRF2 antioxidant pathways, resulting in granulosa cell dysfunction and diminished ovarian reserve.

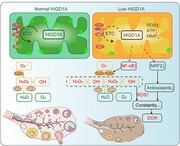

## 1 Introduction

Diminished ovarian reserve (DOR) is a pathological condition that develops progressively with age and is characterized by a variety of clinical symptoms, including a reduction in the quantity and quality of ovarian follicles, a decrease in the level of anti‐Müllerian hormone (AMH), an increase in follicle‐stimulating hormone (FSH), and a decrease in the number of antral follicles (AFC).^[^ ^1^ ^]^ As women age, their ovarian function normally declines and is typically depleted by menopause, which usually occurs between the ages of 49 and 52.^[^ ^2^ ^]^ This process is referred to as physiological DOR. However, approximately 10% of women aged between 20 and 35 may experience ovarian insufficiency (POI), and 1% of these women will progress to premature ovarian failure (POF), which is also referred to as pathological ovarian decline (DOR).^[^ ^3^ ^]^ As more and more women are affected by DOR even infertility,^[^ ^4^ ^]^ it is essential to investigate the mechanisms underlying DOR and develop corresponding intervention strategies.

Oxidative stress (OS) is recognized as a contributor to DOR.^[^ ^5^ ^]^ It occurs when there is an imbalance between the production of reactive oxygen species (ROS) and the antioxidant defense mechanisms, leading to the accumulation of ROS and consequent damage to organs and tissues.^[^ ^6^, ^7^, ^8^, ^9^ ^]^ OS can be caused by factors such as aging, surgery, radiotherapy, and chemotherapy, infection, environmental toxins, and psychological stress,^[^ ^10^, ^11^, ^12^, ^13^, ^14^, ^15^ ^]^ playing a key role in the pathophysiology of ovarian dysfunction. However, the precise mechanisms underlying OS effects on ovarian function remain unclear.

The proper functioning of granulosa cells (GCs) is vital in maintaining female fertility. During folliculogenesis, GCs play a critical role in the biological interaction and communication with oocytes, which is essential for oocyte maturation, ovulation, fertilization, and early‐stage embryonic development.^[^ ^16^ ^]^ However, oxidative stress can lead to damage in GCs, thereby negatively impacting the developmental potential of follicles and ultimately leading to female infertility.^[^ ^17^ ^]^ Therefore, elucidating the molecular regulatory networks underlying OS‐induced GC dysfunction can aid in understanding this process, identifying highly effective molecular targets, and establishing relevant therapies, eventually contributing to the protection of female reproductive health.

Hypoxia‐induced gene domain protein‐1A (*HIGD1A*) is a member of the highly conserved eukaryotic protein family known as the HIG1 domain family. It is a mitochondrial inner membrane protein that plays a crucial role in multiple cell types. *HIGD1A* acts as a positive regulator of cytochrome c oxidase,^[^ ^18^ ^]^ the enzyme that is responsible for the final step of the electron transport chain in mitochondria. Under hypoxic conditions, upregulation of *HIGD1A* leads to increased oxygen consumption and mitochondrial ATP synthesis, promoting cell survival.^[^ ^18^ ^]^ Additionally, HIGD1A facilitates interprotein electron transfer from cytochrome c to cytochrome c oxidase, regulates the fission and fusion of mitochondria, and maintains the balance of the redox system.^[^ ^19^, ^20^, ^21^, ^22^ ^]^

Previous research has demonstrated that high‐fat diet‐induced ROS can induce the expression of *HIGD1A* by upregulating *HIF‐1α* and *PGC‐1α*, thus protecting cells from damage.^[^ ^23^ ^]^ Another study reported that highly expressed *HIGD1A* in pancreatic β cells helps protect cells from apoptosis and increases cell survival rates.^[^ ^24^ ^]^ These findings suggest that *HIGD1A* is crucial in cellular stress response and cytoprotection. However, there is limited research on how HIGD1A participates in the OS process within GCs and the female reproductive system. Future investigations are warranted to elucidate the role of *HIGD1A* in these contexts.

The aim of this study is to comprehensively evaluate the impact of HIGD1A on GCs and ovarian functions, while elucidating the underlying mechanisms of HIGD1A regulating OS in both granulosa cell lines and mouse ovaries. Ultimately, the study intends to identify a novel therapeutic target for OS‐related female infertility.

## 2 Results and Discussion

### 2.1 Higd1a is Highly Expressed in Mouse Ovaries

To clarify the expression pattern of *Higd1a*, we first studied the expression levels of *Higd1a* in various kinds of tissues of ICR mouse. Protein sequence conservation of HIGD1A was analyzed with UGENE software (v40.1), demonstrating that the amino acid sequence of HIGD1A is highly homologous in human, mouse, rat, and pongo abelii (Figure S1A, Supporting Information). Both mRNA and protein levels of *Higd1a* in various tissues were determined. The result of RT‐qPCR exhibited that *Higd1a* mRNA is highly expressed in the ovary, lung, and intestine (Figure S1B, Supporting Information), while the western blot analysis has demonstrated prominent expression of HIGD1A in the intestine, uterus, testis, and ovary (Figure S1C, Supporting Information). These results validated the ovarian localization of *Higd1a*.

To determine the subcellular localization of *HIGD1A* in GCs, we next performed immunofluorescent staining in human granulosa cell lines COV434 and KGN. As shown in Figure S1D (Supporting Information), the distribution pattern of the fluorescent signal suggests that HIGD1A is mainly located in the cytoplasm both in COV434 cell line, KGN cell line, and human GCs in normal conditions, which is consistent with the results from other types of cell lines.^[^ ^25^, ^26^ ^]^ Both of the cell lines were identified with STR analysis and FSHR IF staining (Figure S2, Supporting Information).

### 2.2 HIGD1A is Downregulated in GCs in DOR Patients and 3NP‐Induced OS Models

To clarify the differential expression of *HIGD1A* in GCs between NOR and DOR patients, a total of 145 samples of GCs‐contained human follicular fluid were collected and purified, eventually 121 samples that met the requirements were acquired. The basic information of the participants who qualified for the final analysis is presented in **Table** **1**.

**Table 1:** Clinical characteristics of the enrolled participants.

Characteristic	Overall (*n* = 121)		NOR (*n* = 76)		DOR (*n* = 45)	
	Mean ± SD	*n*	Mean ± SD	*n*	Mean ± SD	*n*
Age	34.74 ± 5.46		32.66 ± 4.92		38.14 ± 4.678	
20–34		66		55		11
≥35		55		21		34
BMI^a)^						
<18.5		9		6		3
18.5–23.9		76		43		33
24–27.9		14		14		0
≥28		6		5		1
Infertility duration^b)^ (years)	3.7 ± 2.9		2.81 ± 1.9	62	4.4 ± 1.6	39
Cause of Infertility						
Primary		62		26		26
Secondary		59		40		19
Basal hormone levels						
FSH (IU/l)	7.483 ± 3.812		6.93 ± 2.84		8.58 ± 5.41	
LH (IU/l)	5.019 ± 2.839		5.22 ± 3.28		4.59 ± 2.18	
Estradiol (pg mL^−1^)	41.13 ± 29.49		44.77 ± 33.87		39.9 ± 21.84	
Progesterone (ng mL^−1^)	0.6856 ± 2.431		0.94 ± 3.06		0.26 ± 0.14	
AMH^c)^ (ng mL^−1^)	2.431 ± 3.253	62	7.21 ± 2.67	24	2.55 ± 1.68	38
AFC^d)^	9.807 ± 6.393	112	13.47 ± 5.2	72	3.9 ± 2.22	40
Ovulation stimulation protocols						
GnRHα long		64		41		23
GnRHα super‐long		5		4		1
GnRHα short		18		12		6
GnRH antagonist		13		10		3
Natural cycle		4		0		4
Minimal stimulation		17		9		8
Laboratory						
IVF		87		60		27
ICSI		34		6		28
Sperm source						
Husband		118		74		44
Donor		3		2		1

^a)^ A total of 105 participants have BMI records;

^b)^ A total of 101 participants have infertility duration records;

^c)^ A total of 101 participants have AMH records;

^d)^ A total of 101 participants have AFC records;

AFC, antral follicle count; AMH, anti‐müllerian hormone; ICSI, intracytoplasmic sperm injection; IVF, in vitro fertilization; SD, standard deviation.

**Figure 1:**
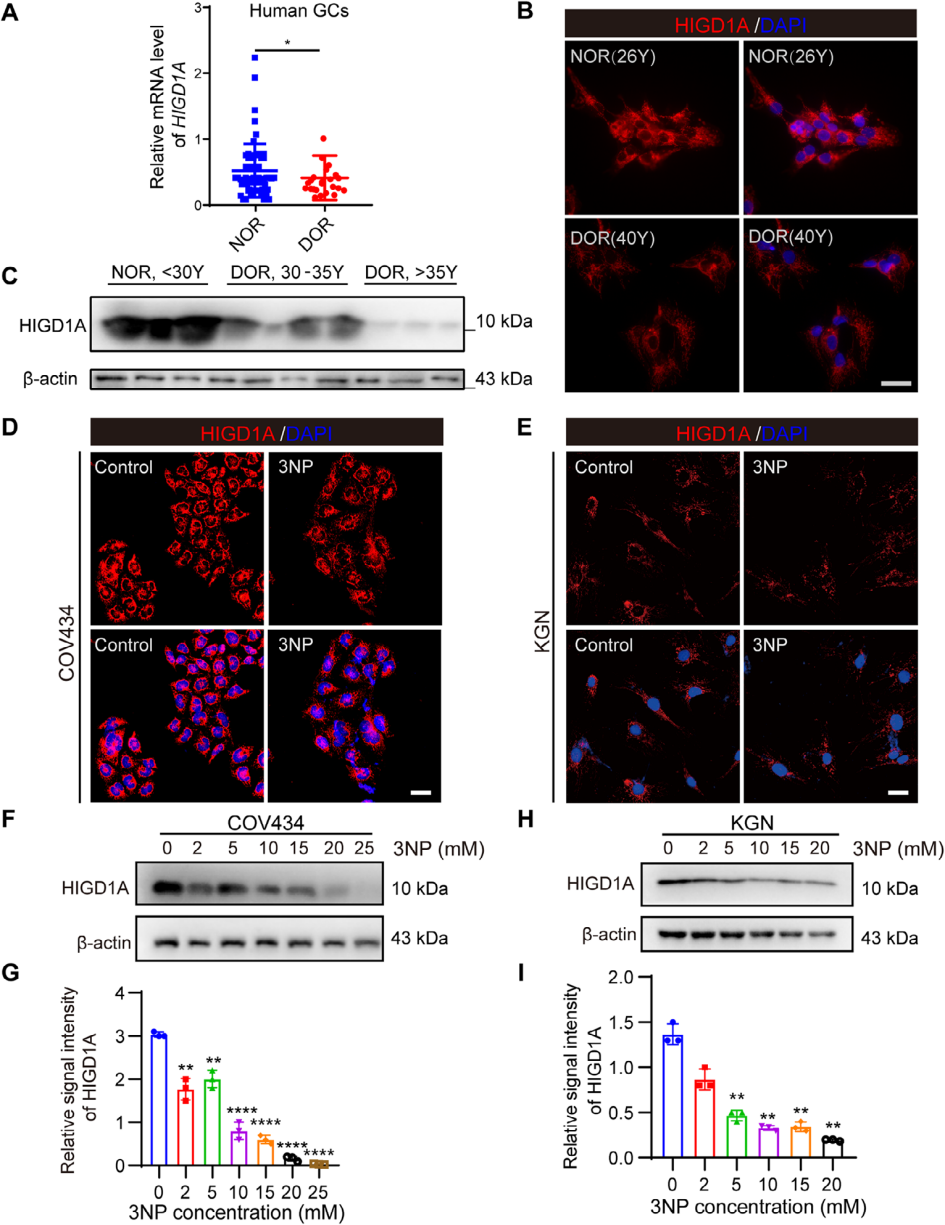
*HIGD1A* is downregulated in GCs in DOR patients and 3NP‐induced OS models. (A) Relative expression levels of *HIGD1A* in GCs of NOR or DOR patients were determined by RT‐qPCR (NOR patients: *n* = 51; DOR patients: *n* = 23). NOR, normal ovarian reserve; DOR, diminished ovarian reserve. (B) Representative images of HIGD1A IF staining in GCs of NOR or DOR patients. (C) Protein levels of HIGD1A in GCs of NOR or DOR patients were determined by western blot. β‐actin served as loading control. (D,E) Representative images of HIGD1A IF staining in 5 mm 3NP‐treated COV434 and KGN cell lines. (F,G) Western blot analysis of HIGD1A protein levels in COV434 cell line upon treatment with different concentration of 3NP. (H,I) Western blot analysis of HIGD1A protein levels in KGN cell line upon treatment with different concentration of 3NP. Scale bar: 40 µm. The experiment was repeated three times and the results were expressed as mean ± standard error and analyzed by *t*‐test (*n* ≥ 3). *p < 0.05, **p < 0.01, ***p < 0.001.

The mRNA and protein levels of *HIGD1A* were analyzed. The results showed that both mRNA and protein levels of *HIGD1A* in DOR patients were significantly lower than those in NOR patients (**Figure** **1**A–C).

We used three different OS inducers, 3‐NP, VK3, and H_2_O_2_ to simulate the OS status in COV434 and KGN cell lines in vitro. The concentrations used were determined according to the IC50 of chemicals (Figure S3A, Supporting Information). The impact of different treatment was evaluated by measuring intracellular ROS levels (Figure S3B–E, Supporting Information). Eventually 3‐NP was selected for subsequent establishment of in vitro OS models. Then, the changes of *HIGD1A* expression upon different treatment were analyzed. Both IF and western blot assays have revealed that 3‐NP treatment resulted in HIGD1A downregulation both in COV434 and KGN cell lines (Figure 1D–H). Furthermore, HIGD1A expression showed a concentration‐dependent downward trend with the increase of 3‐NP treatment concentration (Figure 1F–H), suggesting that *HIGD1A* may play an important role in OS‐induced granulosa cell lines damage.

We next examined the correlation between *Higd1a* expression and mouse age with samples collected from ovarian tissues of 12‐, 24‐, 36‐, 44‐, and 48‐week mice (three mice per group). As shown in Figure S4A,B (Supporting Information), both mRNA and protein levels of *Higd1a* in mouse ovaries gradually declines with age, which was further validated by the result of RNA‐seq analysis from GEO database (GSE154890, Figure S4A, Supporting Information). To further validate the in vivo impact of OS on *Higd1a* expression, we established and evaluated OS models in ICR mice. Three different chemicals, VK3, 3‐NP and CTX were all administered through intraperitoneal injection, after which mouse estrous cycles (Figure S5A, Supporting Information), ovarian index (Figure S5B, Supporting Information), serum hormone levels (Figure S5C–F, Supporting Information), oxidant and antioxidant levels (Figure S5G, Supporting Information) were analyzed for model evaluation. Finally, 3‐NP was selected to establish in vivo OS models. In accordance with in vitro experiments, 3‐NP administration also led to decreased *Higd1a* levels in mouse ovaries (Figure S4C–E, Supporting Information), indicating the close relationship between *Higd1a* and OS‐induced ovarian injuries in vivo.

### 2.3 *HIGD1A* Regulates Proliferation, Cell Cycle, and Cell Apoptosis of GC Cell Lines

**Figure 2:**
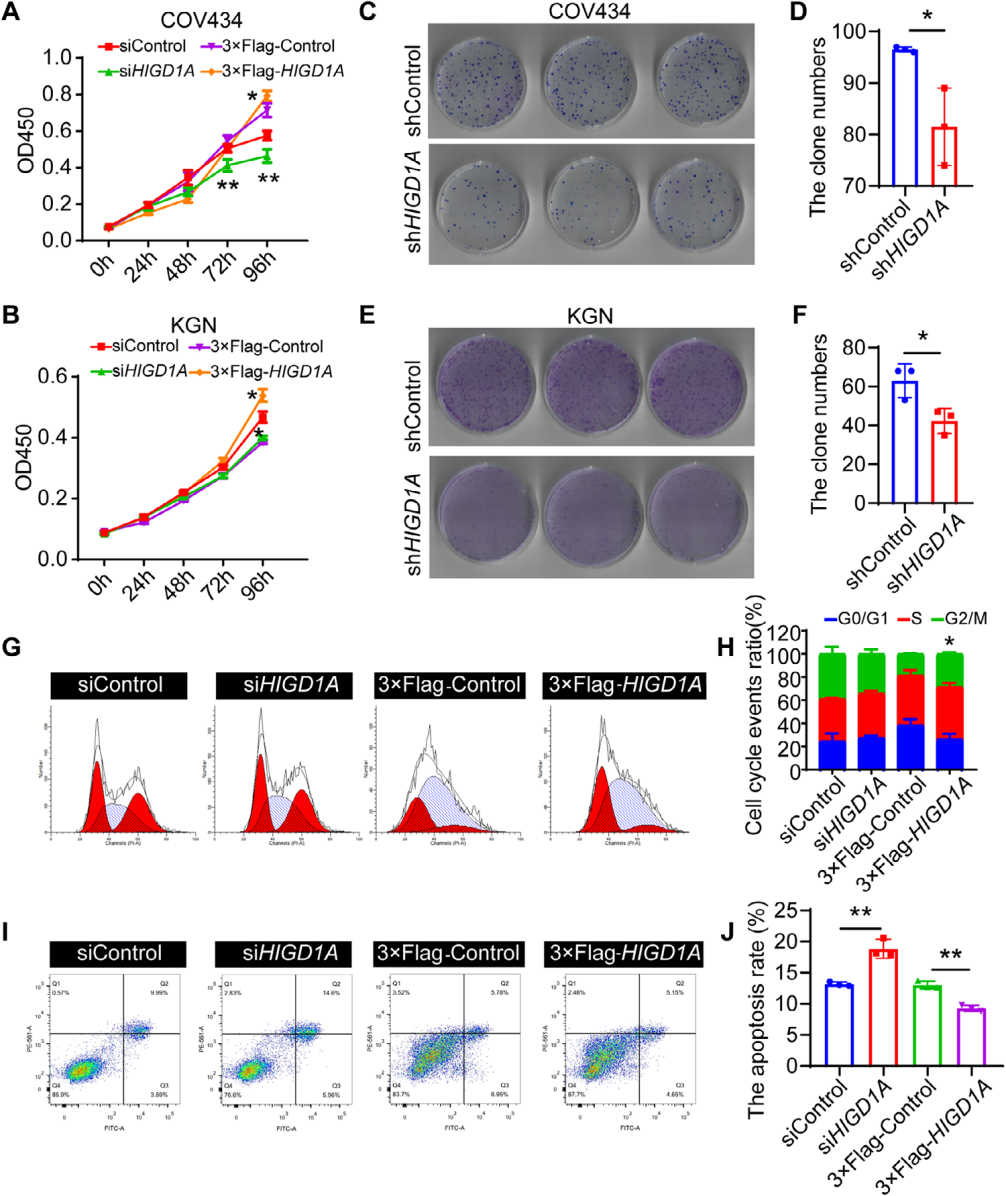
*HIGD1A* regulates proliferation, cell cycle, and cell apoptosis of GC cell lines. (A,B) CCK‐8 analysis revealing the impact of *HIGD1A* knockdown or overexpression on cell viability in COV434 and KGN cell lines (*n* = 12 per group). (C,D) Representative graphs and quantitative analysis of colony formation assays performed in *HIGD1A*‐depleted COV434 cell line (*n* = 12 per group). (E,F) Representative graphs and quantitative analysis of colony formation assays performed in *HIGD1A*‐depleted KGN cell line (*n* = 12 per group). (G,H) PI‐labeled flow cytometry showing the impact of *HIGD1A* knockdown or overexpression on cell cycle phase distribution in COV434 cell line. (I,J) The apoptosis rate of COV434 cell line was determined by Annexin V/PE‐labelled flow cytometry. The experiment was repeated three times and the results were expressed as mean ± standard error and analyzed by *t*‐test (*n* ≥ 3). *p < 0.05, **p < 0.01.

We next investigated the effects of *HIGD1A* on GC functions via loss‐ and gain‐of‐function experiments. The transfection efficiency in COV434 cell lines was identified with a cy3‐labeled siRNA, while the knockdown and overexpression efficiency were determined at both transcriptional and translational levels (Figure S6, Supporting Information). Next, we evaluated the cell viability via the CCK‐8 assay. *HIGD1A* knockdown inhibited cell viability of COV434 and KGN cell lines at 48 h and 72 h after transfection, respectively (**Figure** **2**A,B). In contrast, *HIGD1A* overexpression enhanced cell viability both in COV434 and KGN cell lines after transient *HIGD1A* overexpression. We then developed *HIGD1A*‐shRNA stably transfected COV434 and KGN cell lines with the lentivirus shRNA knockdown system to analyze the impact of *HIGD1A* knockdown onGC cell lines proliferation. As expected, *HIGD1A*‐depleted cells formed fewer colonies compared to cells from the control group, indicating *HIG1DA* knockdown significantly impaired the GC cell lines growth ability (Figure 2C,E). Since cell cycle and apoptosis are both key regulators of cell proliferation, flow cytometry was performed to evaluate the cell cycle phase distribution and cell apoptotic rate. As shown in Figure 2G‐J, *HIGD1A* knockdown did not obviously affect cell cycle and apoptosis of GC cell lines, whereas the ectopic expression of *HIGD1A* facilitates cell cycle progression and alleviated the GC cell lines apoptosis.

### 2.4 *HIGD1A* Depletion Aggravates Cellular Senescence and Impairs Steroid Hormone Synthesis in GC Cell Lines

**Figure 3:**
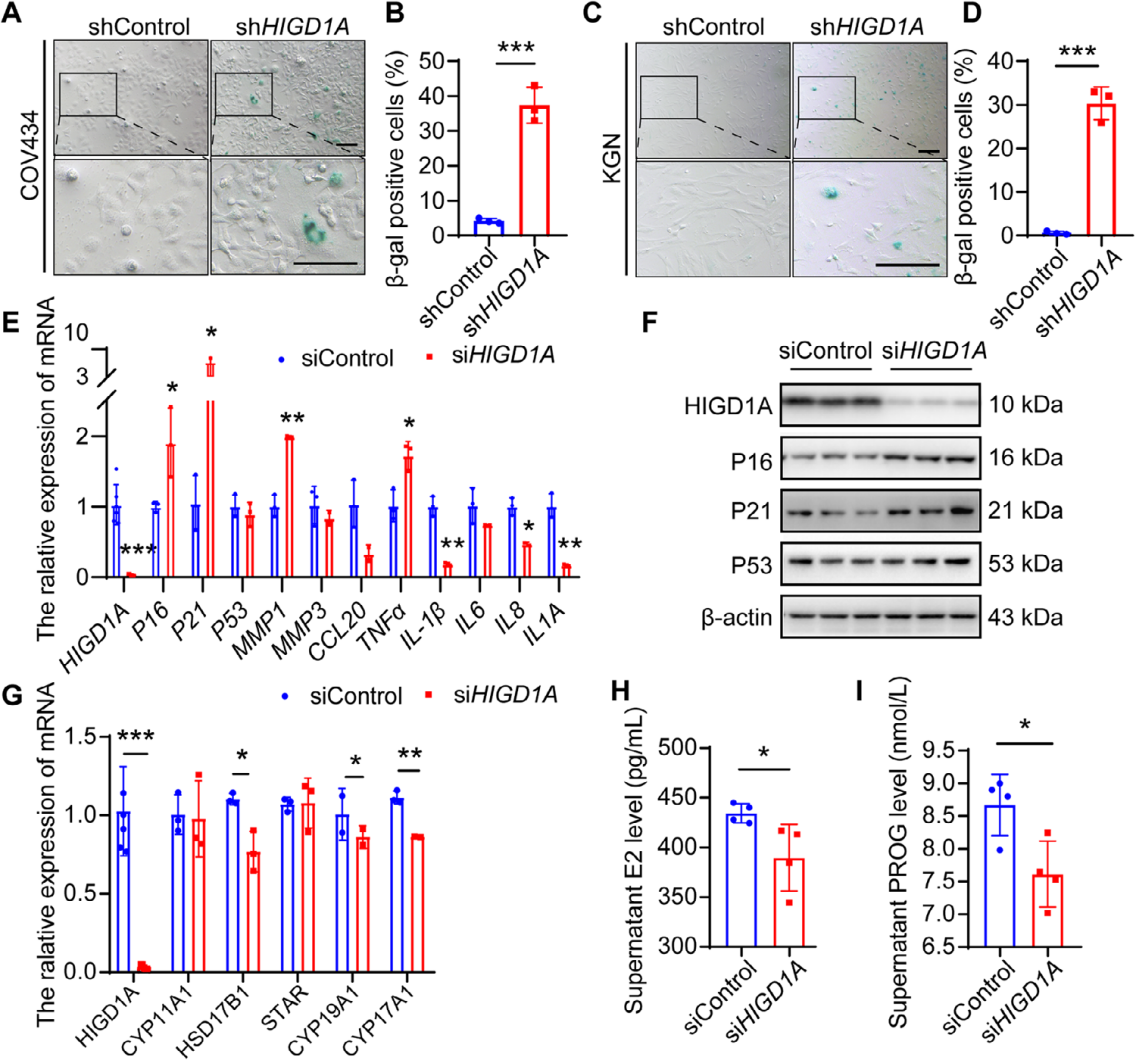
*HIGD1A* regulates cellular senescence and steroid hormone synthesis in GC cell lines. (A–D) Representative images and quantitative analysis of SA‐β‐gal staining in *HIGD1A‐*depleted COV434 and KGN cell lines. (E,F) RT‐qPCR and western blot analysis of mRNA and protein levels of *HIGD1A* and cellular senescent markers. (G) RT‐qPCR analysis of mRNA levels of hormone synthesis‐related genes. (H,I) Supernatant estradiol (E2) and progesterone (PROG) levels were analyzed by ELISA (*n* = 5). The experiment was repeated three times and the results were expressed as mean ± standard error and analyzed by *t*‐test (*n* ≥ 3). *p < 0.05, **p < 0.01, ***p < 0.001.

We then measured the impact of *HIGD1A* knockdown on cellular senescence which is under the regulation of cell cycle and proliferation to a large extent. SA‐β‐gal staining was performed in *HIGD1A*‐knockdowned COV434 and KGN cell lines, and quantification was done by counting the β‐gal‐positive cells. As a result, elevated positive rates, which demonstrated an exacerbated cellular senescent status, were observed in both cell lines after *HIGD1A* knockdown (**Figure** **3**A–D). The changes of cellular senescent markers, *p16*, *p21*, and *p53* upon *HIGD1A* knockdown were also investigated, with the mRNA and protein levels of *p16* and *p21* drastically increased while *P53* not significantly affected (Figure 3E,F). Furthermore, we analyzed the senescence‐associated secretory phenotype (SASP) by measuring the mRNA levels of several typical SASP factors. Among all the examined factors, *MMP1* and *TNF‐α* were up‐regulated, while *IL‐1β*, *IL‐8* and *IL1A* were inhibited, and the remaining factors were not significantly changed (Figure 3E), indicating aggravated GC cell lines senescence induced by *HIGD1A* knockdown is not SASP‐related. Synthesis of steroid hormones is one of the important physiological functions of GCs. RT‐qPCR analysis revealed that *HIGD1A* knockdown inhibited expression of several hormone‐synthesis‐related factors (Figure 3G). This result may help to explain the decreased estradiol and progesterone levels in cell culture supernatant (Figure 3H,I). In summary, *HIGD1A* depletion led to GC cell lines  damage in multiple aspects including cell viability, proliferation, cellular senescence, and steroid hormone synthesis, eventually resulting in GC cell lines dysfunction.

### 2.5 *HIGD1A* Modulates Mitochondrial Functions and Oxidative Stress in GC Cell Lines

**Figure 4:**
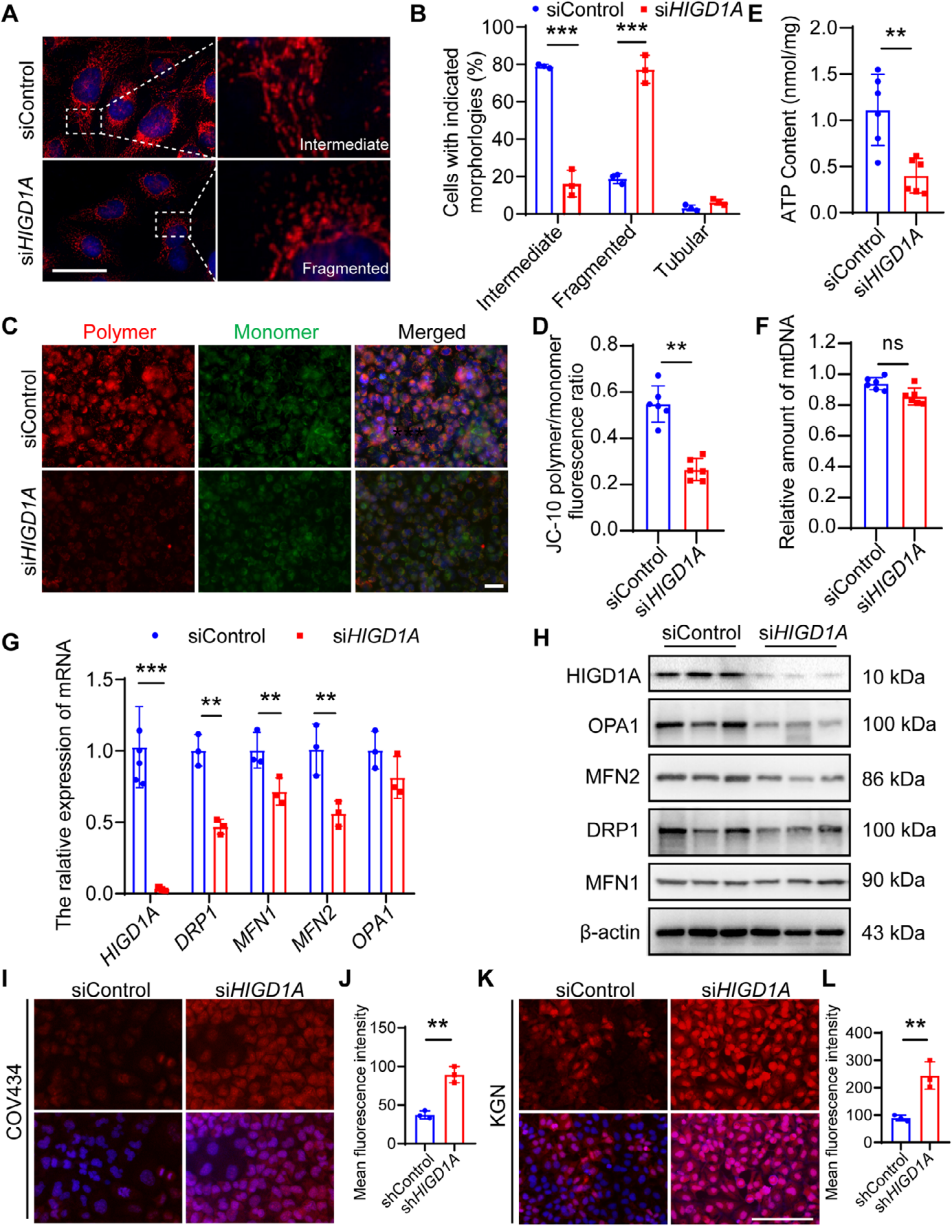
*HIGD1A* regulates mitochondrial functions and oxidative stress in GC cell lines. (A,B) Representative images and classification statistics of mitochondrial morphology visualized by Mito‐tracker Red. (C,D) Mitochondrial membrane potential in COV434 cell line was analyzed by JC‐10 assay. (E,F) Changes in intracellular ATP content and mtDNA copy numbers in COV434 cell line were measured. (G,H) The mRNA and protein levels of mitochondrial fission and fusion‐related genes were determined by RT‐qPCR and western blot analysis. β‐actin served as loading control. (I–L) Representative images and quantitative analysis of intracellular ROS levels in COV434 and KGN cell lines. The experiment was repeated three times and the results were expressed as mean ± standard error and analyzed by *t*‐test (*n* ≥ 3). **p < 0.01, ***p < 0.001.

Given that *HIGD1A* serves as a key component of the mitochondrial respiratory chain, we next investigated the impact of *HIGD1A* knockdown on mitochondrial functions. The mitochondrial probe, Mito‐Tracker red, was used to observe the mitochondrial morphology. Previous studies have categorized mitochondria into three distinct morphological types: tubular, intermediate, and fragmented.^[^ ^18^ ^]^ As shown in **Figure** **4**A,B, *HIGD1A* knockdown increased proportion of fragmented mitochondria, while the predominant morphotype in the control group is the intermediate, suggesting that *HIGD1A* depletion impaired the fusion of mitochondria. To further validate this conclusion, we analyzed the expression levels of several genes regulating mitochondria fusion and fission via RT‐qPCR and western blot. As a result, the decreased mRNA level of *MFN1* and *MFN2* as well as the decreased protein level of MFN2 and OPA1 collectively evidenced that *HIGD1A* knockdown hindered the fusion of mitochondria (Figure 4G,H). We then evaluated the mitochondrial membrane potential (MMP) using a JC‐10 assay kit. The decreased polymer‐monomer ratio in the *HIGD1A*‐knockdown group indicated the decline in MMP (Figure 4C,D), which is consistent with the reduced ATP level (Figure 4E). The mtDNA copy number, however, was not significantly affected (Figure 4F).

Since mitochondria are a main source of intracellular ROS and mitochondrial dysfunction may result in excessive ROS generation, imbalance of redox system and subsequent OS, we next investigated whether *HIGD1A* knockdown induced OS in GC cell lines. ROS detection and relative quantification was achieved by dichloro‐dihydrofluorescein diacetate (DCFH‐DA) assay, showing that *HIGD1A* knockdown increased the generation of intracellular ROS (Figure 4I,K).

### 2.6 Transcriptome Profiling Reveals the Impact of *HIGD1A* Knockdown on GC Cell Line

**Figure 5:**
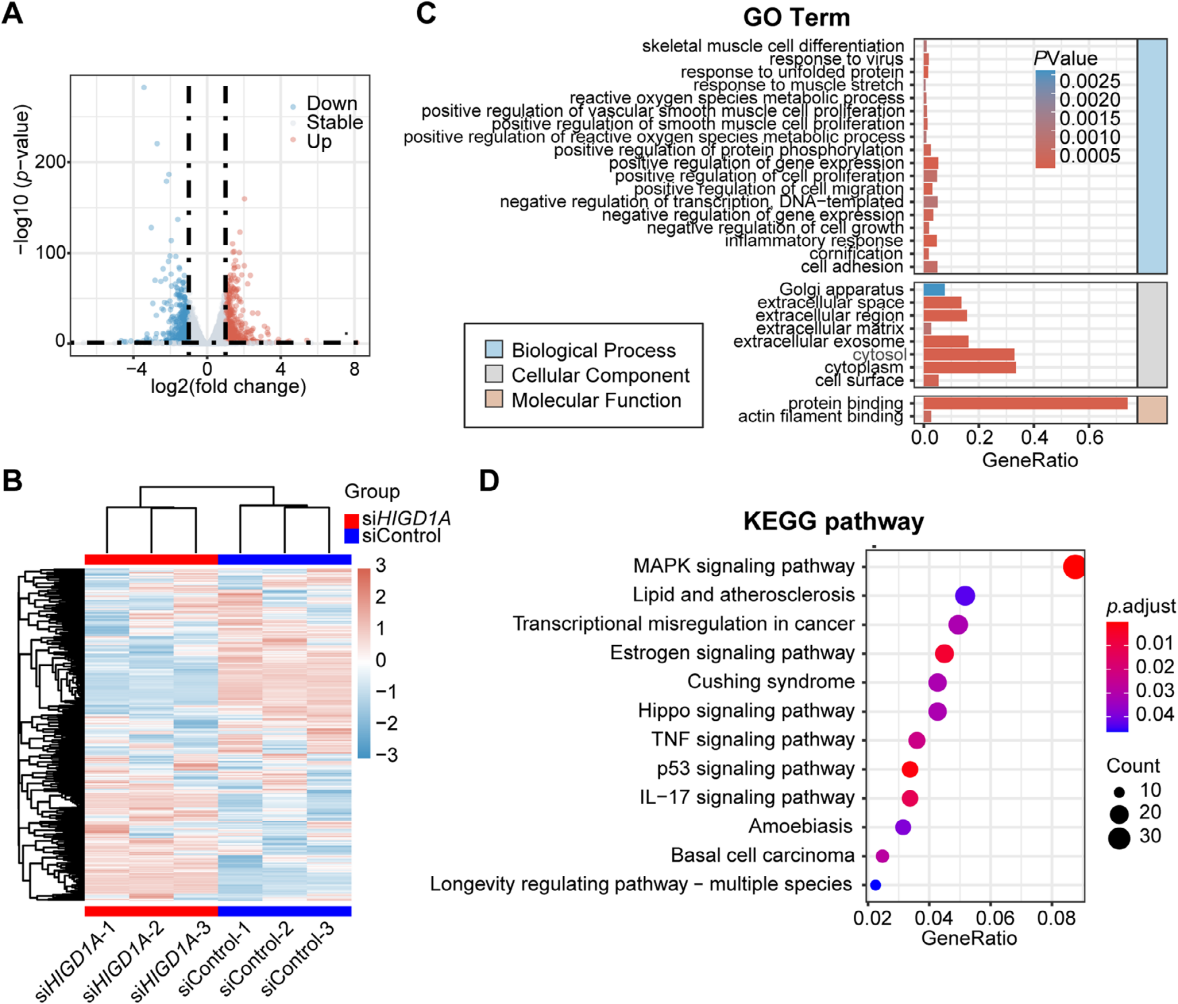
Transcriptome profiling revealing the impact of *HIGD1A* knockdown on GC cell lines. (A) Volcano plot showing differentially expressed genes upon *HIGD1A* knockdown. (B) Expression heatmap showing difference of gene counts profiled by RNA‐seq. Z‐score serves as the color key. (C) Bar blot showing the enriched GO terms. (D) Dot plot showing the enriched KEGG pathways. GeneRatio is defined as (count of core enrichment genes)/ (count of pathway genes).

To further identify the specific molecular regulatory networks participating in *HIGD1A*‐knockdown‐meditated GC cell line damage, we performed RNA‐seq in COV434 cell line transfected with *HIGD1A*‐directed siRNA or its negative control. The volcano plot demonstrates the 994 differentially expressed genes (cut‐off values: |log2(fold change)| ≥ 1 and adjusted *p*‐value < 0.05) upon *HIGD1A* knockdown, with 506 upregulated genes and 488 downregulated genes (**Figure** **5**A). The heatmap displays the relative expression levels of 1200 genes with the most significant inter‐group difference (Figure 5B). Clustering analysis indicates that *HIGD1A* knockdown has a relatively stable effect on the cellular transcriptome profile.

The result of GO and KEGG enrichment analysis were illustrated with a bar plot and a dot plot, respectively. A total of 28 GO terms with *p*‐value < 0.0025 were enriched in the analysis, with 18 biological process terms, 8 cellular component terms, and 2 molecular function terms (Figure 5C). Twelve KEGG signaling pathways, including MAPK signaling pathway, estrogen signaling pathway, Hippo signaling pathway, TNF signaling pathway, and p53 signaling pathway were enriched in the KEGG enrichment analysis (Figure 5D), further validating previous results that *HIGD1A* knockdown impaired GC cell line functions and led to aggravated cellular senescence.

### 2.7 *HIGD1A* Overexpression Rescues 3‐NP‐Induced GC Cell Line Damage

**Figure 6:**
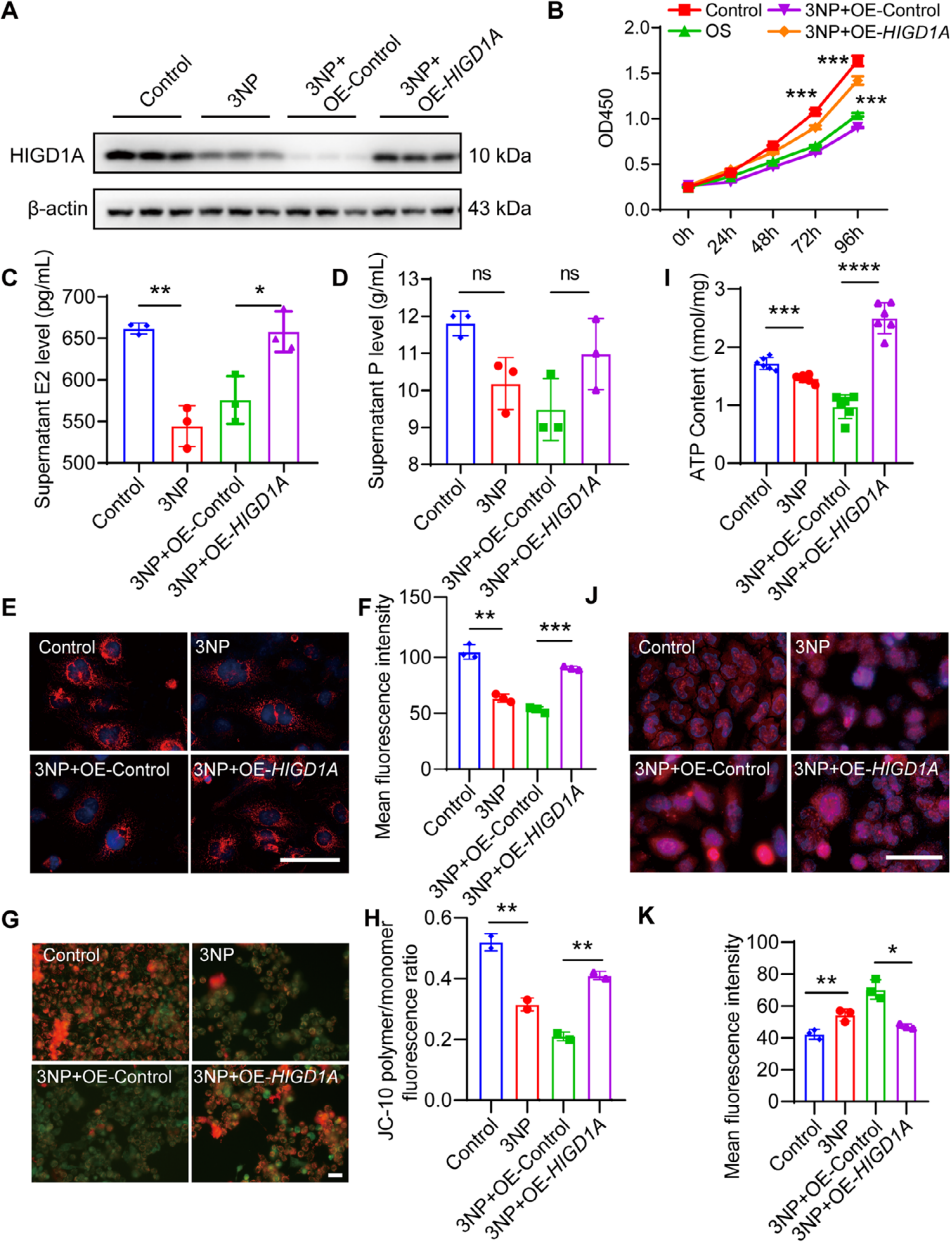
*HIGD1A* overexpression rescues 3‐NP‐induced GC cell line damages. (A) Western blot analysis of HIGD1A expression after 3NP treatment and HIGD1A overexpression. (B) Cell viability of COV434 cell line was determined by CCK‐8 assay (*n* = 12 per group). (C,D) Supernatant estradiol and progesterone levels of COV434 cell line were determined by ELISA. (E,F) Mitochondrial distribution and quantitative analysis in COV434 cell lin were determined by Mito‐Tracker Red staining. (G,H) Mitochondrial membrane potential in COV434 cells was analyzed by JC‐10 assay. (I) Intracellular ATP levels in COV434 cells were measured. (J,K) Representative images and quantitative analysis of intracellular ROS levels visualized by DCFH‐DA probe. The experiment was repeated three times and the results were expressed as mean ± standard error and analyzed by *t*‐test (*n* ≥ 3). *p < 0.05, **p < 0.01, ***p < 0.001.

After revealing the role of *HIGD1A* in regulating GCs functions, we treated *HIGD1A*‐overexpressing cells with the OS‐inducer 3‐NP to explore the anti‐OS effect of *HIGD1A* in GC cell line. The protein levels of HIGD1A in COV434 cell line after *HIGD1A* overexpression and 3‐NP treatment were first analyzed by western blot (**Figure** **6**A). The cell viability was evaluated via the CCK‐8 assay. As shown in Figure 6B, *HIGD1A* overexpression partially reversed 3‐NP‐induced decrease in cell viability. We then analyzed the steroid hormone synthesis capacity of COV434 cell line by ELISA. The results indicate that *HIGD1A* overexpression rescued the 3‐NP‐induced reduction of E2 levels (Figure 6C). However, both 3NP treatment and *HIGD1A* overexpression (with 3NP treatment) did not significantly affect the secretion of progesterone in COV434 cell line (Figure 6D).

Next, we analyzed the changes of mitochondrial functions and the levels of OS. As shown in Figure 6E–H, *HIGD1A* overexpression increased the mitochondrial density and MMP in 3‐NP‐treated cells. Similarly, the intracellular ATP content also exhibited an obvious upward trend in *HIGD1A*‐overexpressed cells (Figure 6I). In terms of OS levels, *HIGD1A* overexpression efficiently alleviated 3‐NP‐induced ROS overloading (Figure 6J,K). Taken together, these results suggest that HIGD1A facilitates resistance to OS damage by protecting mitochondrial functions and reducing ROS generation, which helps to restore the hormone synthesis capacity in GC cell line.

### 2.8 *HIGD1A* Regulates NF‐κB/SOD2 Pathway in GC Cell Line

**Figure 7:**
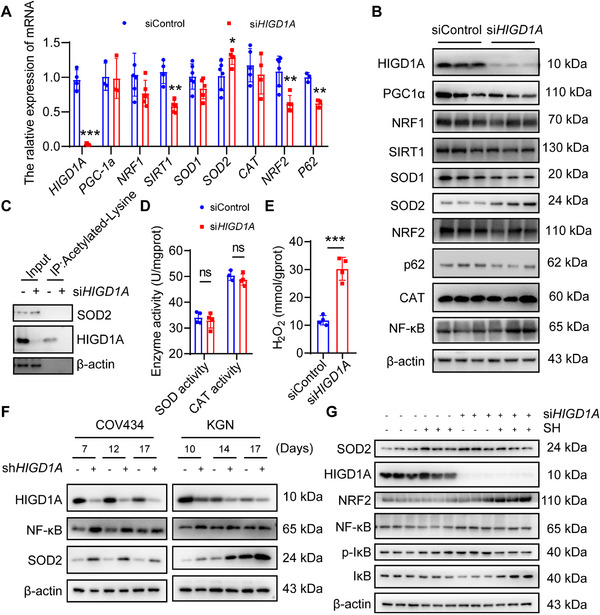
*HIGD1A* regulates NF‐κB/SOD2 pathway in GC cell lines. (A,B) RT‐qPCR and western blot analysis of mRNA and protein levels of oxidative stress‐related genes in COV434 cells. (C) Changes in the acetylation level of SOD2 upon *HIGD1A* knockdown was determined by acetylated‐lysine immunoprecipitation. (D) Enzyme activities of SOD and CAT in COV434 cells were measured. (E) Changes in intracellular H_2_O_2_ levels upon *HIGD1A* knockdown were determined. (F) Representative western blot analysis of NF‐Κb and SOD2 protein levels upon *HIGD1A* knockdown. β‐actin served as loading control. (G) Western blot analysis of indicated protein levels upon *HIGD1A* depletion and treatment of NF‐κB inhibitor SH. β‐actin served as loading control. SH, sinomenine hydrochloride. The experiment was repeated three times and the results were expressed as mean ± standard error and analyzed by *t*‐test (*n* ≥ 3). *p < 0.05, **p < 0.01, ***p < 0.001.

We next tried to unveil by which means HIGD1A plays a role in the OS‐related process in GCs. We first performed RT‐qPCR and western blot to test whether the mRNA and protein levels of OS‐related factors change upon *HIGD1A* depletion. As shown in **Figure** **7A** and 7B, several anti‐OS factors, including SIRT1, PGC1α, NRF1, NRF2 and SOD1 were downregulated after *HIGD1A* knockdown, while the levels of SOD2 and NF‐κB obviously elevated.

Previous studies revealed that SOD2 is a typical anti‐OS factor normally being downregulated under OS exposure,^[^ ^27^, ^28^ ^]^ making SOD2 upregulation an intriguing result that worth further exploration. Since SOD2 acetylation contributes to SOD2 deactivation,^[^ ^29^ ^]^ we detected the acetylation level of SOD2 by immunoprecipitation but did not find any significant changes (Figure 7C), indicating that SOD2 was not regulated by acetylation modification. SODs are a group of enzymes that catalyze the dismutation of superoxide anion to O_2_ and H_2_O_2_.^[^ ^30^ ^]^ To clarify whether SOD2 upregulation is involved in meditating *HIGD1A*‐knockdown‐induced OS by promoting H_2_O_2_ production, we examined the intracellular H_2_O_2_ levels and enzyme activities of SOD (including SOD1 and SOD2) and CAT (an enzyme that catalyzes the reaction by which H_2_O_2_ decomposes to form water and oxygen). As shown in Figure 7D and 7E, *HIGD1A* knockdown drastically increased the amount of cellular H_2_O_2_ without affecting enzyme activities of SOD and CAT. Since SOD1 downregulation and SOD2 upregulation have been observed in previous studies, abnormally increased SOD2 might be responsible for the H_2_O_2_ overproduction and, in consequence, *HIGD1A*‐knockdown‐induced redox imbalance in COV434 cell line.

NF‐κB is a well‐studied redox‐sensitive transcription factor regulating the expression of hundreds of target genes including SOD2. The upregulation of NF‐κB after *HIGD1A* knockdown suggests that the enhanced SOD2 expression might be attributed to the NF‐κB signaling pathway. To verify this hypothesis, we first examined the expression of NF‐κB and SOD2 in *HIGD1A*‐depleted COV434 and KGN cell lines and observed consistent upregulations of NF‐ΚB and SOD2 (Figure 7F). Next, we treated *HIGD1A*‐knockdowned COV434 cell line with NF‐κB inhibitor sinomenine hydrochloride (SH), and performed western blot analyses to determine the protein levels of factors in NF‐κB signaling pathway. The results showed that the increased SOD2 expression in the si*HIGD1A* group was partially reversed by NF‐κB inhibitor (Figure 7G), which is in support of the hypothesis that *HIGD1A* regulates SOD2 expression via NF‐κB signaling pathway.

### 2.9 *Higd1a* Overexpression Rescues Ovarian Function in Mice

**Figure 8:**
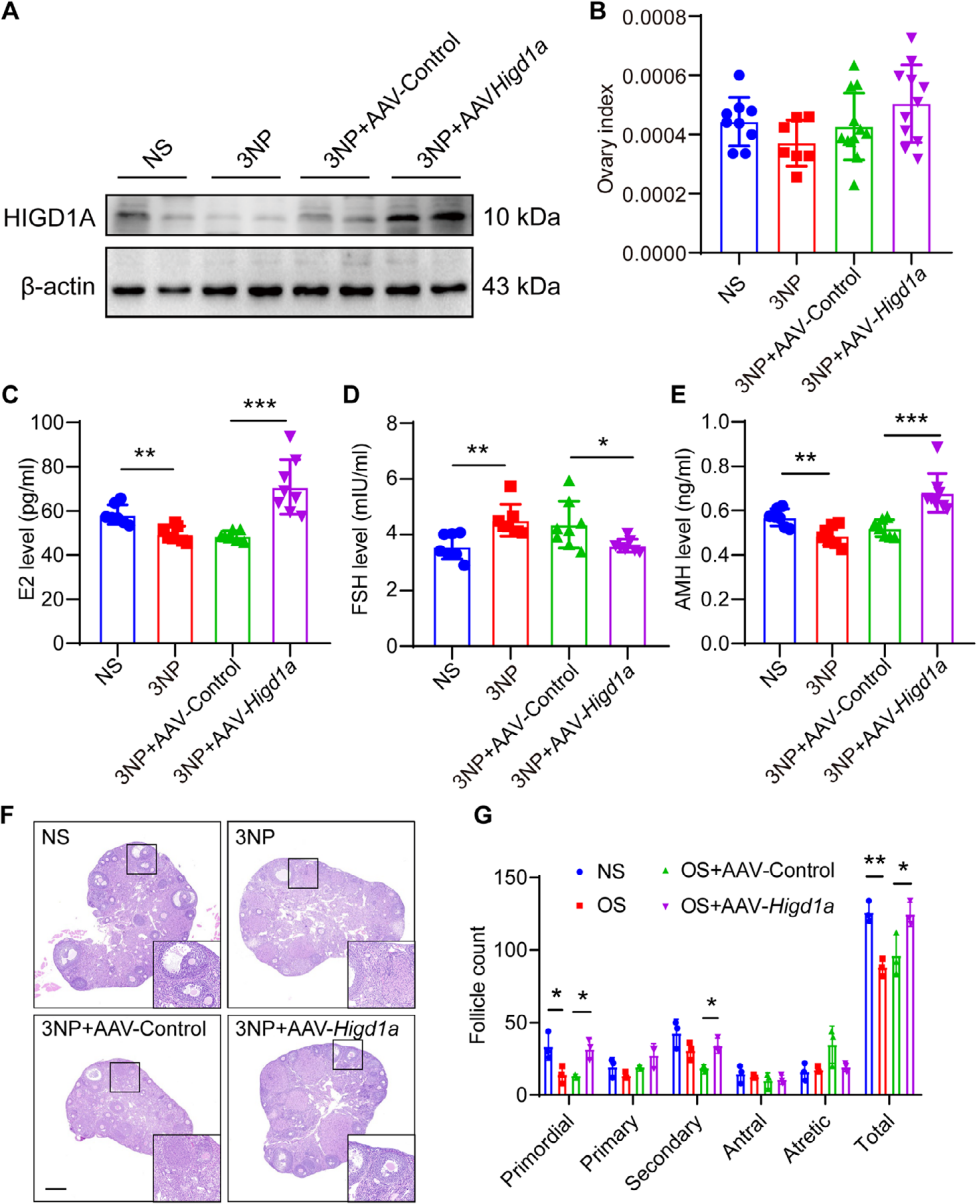
*Higd1a* overexpression rescues ovarian function in mice. (A) HIGD1A protein levels of mouse ovaries after 3NP administration and AAV‐mediated HIGD1A overexpression were determined by western blot analysis. β‐actin served as loading control. (B) Ovary index was recorded after 3NP and AAV administration (*n* = 12 per group). (C–E) The levels of E2, FSH, and AMH in serum samples were determined by ELISA (*n* = 12 per group). (F,G) Representative images of HE staining showing ovarian morphology of each group. Follicle numbers of all developmental stages were counted. The experiment was repeated three times and the results were expressed as mean ± standard error and analyzed by *t*‐test (*n* ≥ 3). *p < 0.05, **p < 0.01, ***p < 0.001.

After confirming the involvement of *Higd1a* in the anti‐OS process in vitro, we next tried to characterize its impact on mouse ovaries. The OS model of mouse ovaries was established as previously described. Adeno‐associated virus (AAV) vectors harboring CDS region of *Higd1a* and ZsGREEN were constructed for subsequent AAV production. AAV particles were administered via ovarian local injection. The overexpression efficiencies of AAV‐*Higd1a* in vitro and in vivo were validated in HEK293T cell line and mouse ovaries, respectively (**Figure** **8A** and Figure S7A,B, Supporting Information).

Body weights were recorded daily during 3‐NP administration, while ovarian weights were measured immediately after euthanasia. The results showed that 3‐NP induced a decrease in body weight, and the ovarian index (defined as ovarian weight/body weight) was effectively rescued by *Higd1a* overexpression in mice that received AAV‐*Higd1a* administration. (Figure 8B and Figure S7C, Supporting Information). We next evaluated the effect of *Higd1a* on hormone secretion by measuring the level of serum E2, FSH, and AMH. As shown in Figure 8C–E, hormone disorders in 3‐NP‐treated mice (manifested as increased FSH levels and decreased E2 and AMH levels) were alleviated by *Higd1a* overexpression, which is consistent with the result of in vitro experiments. Since the hypothalamic‐pituitary‐gonadal axis is a major signaling pathway controlling estrous cycles, we recorded the changes in the estrous cycle of mice in each group and visualized the result with curve plots. Compared with the “3NP+AAV‐Control” group, the estrous cycle progression of the mice in the “3NP+AAV‐ *Higd1a*” group basically returned to a normal state (Figure S8A, Supporting Information). Consistently, the result of follicle counts also validates the beneficial effect of *Higd1a* on ovarian functions, with primordial, primary as well as total follicle counts obviously increased after *Higd1a* overexpression (Figure 8F,G).

The anti‐OS capacity of *Higd1a* in vivo was evaluated by measuring SOD and CAT enzyme activities and MDA levels. As a result, *Higd1a* overexpression not only enhanced SOD and CAT activities, but also inhibited the MDA elevation (Figure S8B–D, Supporting Information), further evidencing that *Higd1a* overexpression can rescue the 3‐NP induced ovarian dysfunction.

## 3 Discussion

OS is a state of imbalance in the redox system, with excessive oxidants overwhelming the antioxidant defense and causing damage to cells and tissues. Originating from various sources such as environmental pollution, radiation, drugs, and mental stress, OS is one of the culprits of many pathological processes and diseases including age‐related dysfunctions and female reproductive disorders.^[^ ^10^, ^31^ ^]^ Consequently, unveiling the mechanisms underlying OS damage can be helpful for further interpreting female infertility and developing intervention measures and therapies correspondingly.

As a component of mitochondria, the role of HIGD1A in regulating mitochondrial functions and OS processes has been elucidated in various cell types and organs,^[^ ^20^, ^23^, ^26^ ^]^ while its participation in female reproductive system is barely studied. HIGD1A is normally located in the cytoplasm and more specifically, on the inner membrane of mitochondria, interactswith cytochrome c to maintain the function of the mitochondrial respiratory chain. The expression of HIGD1A, which is closely correlated with stress conditions such as hypoxia or glucose deprivation, largely depends on the binding of hypoxia‐inducible transcription Factor HIF‐1α to its promoter region.^[^ ^32^ ^]^ However, in some certain conditions HIGD1A can be induced in a HIF‐1α‐independent manner, inhibiting oxygen consumption and reducing cellular ROS levels.^[^ ^32^ ^]^

In this study, we have characterized the localization and basic expression level of HIGD1A in GCs both in vitro and in vivo. HIGD1A expression in granulosa cells was not only downregulated in DOR patients and 3NP‐induced OS models, but also negatively correlated with mouse ages. However, CTX treatment led to obvious upregulation and nucleus translocation of HIGD1A (Figure S9A, Supporting Information). This phenomenon consists with the results of previous studies that HIGD1A located in the nucleus was significantly increased under serious stress conditions, which is presumed to be associated with DNA damage response.^[^ ^25^ ^]^ HIF‐1α is a key upstream regulator of HIGD1A expression. However, we did not observe HIF‐1α expression in ovarian follicles either in the control or OS group (Figure S9B, Supporting Information), indicating that *HIGD1A* expression in ovarian OS models was not under the control of HIF‐1α. In contrast, in H_2_O_2_‐treated COV434 cell line, both HIF‐1α and HIGD1A were upregulated, demonstrating H_2_O_2_ regulates HIGD1A expression via the typical HIF‐1α‐dependent pathway. We additionally examined the expression of HIF‐1α and HIGD1A under hypoxic conditions in our experimental models. Immunofluorescence and Western blot analysis revealed that hypoxia activated this pathway in both mouse granulosa cells and cultured cell lines. However, the expression of HIF‐1α was undetectable under non‐hypoxic conditions. Based on these preliminary observations, we are aim to further explore the potential role of the HIF‐1α/HIGD1A axis in the pathogenesis of hypoxia‐related ovarian diseases, such as ovarian torsion and ovarian tissue transplantation.

The above analysis indicates that different treatments can affect HIGD1A expression through different pathways. H_2_O_2_ activates the HIF‐1 pathway to enhance HIGD1A expression. CTX promotes HIGD1A expression and nuclear translocation through DNA damage. VK3 causes a decrease in the HIGD1A level by inducing oxidative damage, while 3‐NP mediates the significant downregulation of HIGD1A by disrupting the mitochondrial electron‐transfer chain. These data collectively suggest that HIGD1A may serve as an important regulator in ovarian OS damage.

Our study also elucidated the impact of *HIGD1A* depletion on GC cell lines. *HIGD1A* knockdown resulted in increased ROS generation, impaired mitochondrial functions, and mitochondrial fusion disorders. Specifically, *HIGD1A* depletion reduced the levels of several anti‐OS factors including SIRT1, PGC1α, NRF1, NRF2, SOD1, and p62. NRF2 is a key regulator of redox homeostasis. It modulates transcriptional activation of genes related to antioxidant biosynthesis, remodeling cellular metabolism and gene expression along with other stress response pathways. Furthermore, NRF2 functions as a cytoprotective factor by activating p62. Thus, downregulation of NRF2 and p62 upon *HIGD1A* knockdown suggests the diminished anti‐OS capacity of GC cell lines. SIRT1/PGC1α also serves as one of the crucial pathways regulating OS response. It has been reported that inhibition of this pathway aggravated neuron apoptosis, reduced SOD enzyme activity and increased malondialdehyde concentration after cerebral ischemia in rats.^[^ ^33^ ^]^ In addition, SIRT1/3 can induce deacetylation of PGC‐1α, upregulating NRF1 and NRF2 to participate in mitochondrial biosynthesis and consequently, protecting cells.^[^ ^34^ ^]^ In this study, *HIGD1A*‐depletion‐induced downregulation of SIRT1/PGC1α pathway may also be part of the mechanism underlying OS‐induced cell damage.

*HIGD1A* knockdown also led to enhanced SOD2 expression in GC cell lines. As a mitochondrial antioxidant enzyme, increased SOD2 theoretically refers to an alleviated OS status, which, however, is clearly not consistent with the reality.^[^ ^35^ ^]^ There, we considered whether SOD2 was inactivated by acetylation modifications. We performed IP to detect the level of acetylated SOD2 and no significant increase was observed, indicating that SOD2 was not affected by acetylation modifications. In order to further clarify the mechanism of OS induced by *HIGD1A* knockdown, we measured the enzyme activities of SOD (including SOD2 and SOD1) and CAT in GC cell lines. It turns out that *HIGD1A* knockdown did not affect the enzyme activities of SOD and CAT. Combined with the result that the protein level of CAT did not obviously change, we speculate that the increased SOD2/CAT ratio eventually leads to the deterioration of cellular OS because the overproduced H_2_O_2_ (catalyzed by SOD2) cannot be timely decomposed into water and oxygen by CAT. This hypothesis was further validated by the subsequent detection of intracellular ATP levels. All these data suggest that SOD2 elevation is a cause rather than a consequence of OS in a 3‐NP induced OS model.

Previous studies revealed that OS exposure often leads to aggravated cellular senescence. We analyzed the expression levels of senescence markers p16, p21, and p53.^[^ ^36^ ^]^ The results showed that p16 and p21 were obviously upregulated in *HIGD1A*‐depleted cells, further evidencing the correlation between OS and cellular senescence.^[^ ^37^ ^]^ Senescent cells often tend to secrete several chemokines, growth factors, pro‐inflammatory cytokines and proteolytic enzymes,^[^ ^38^ ^]^ which is known as the senescence‐associated secretory phenotype (SASP). SASPs are distinct phenomena regulated by different pathways including IL‐1 signaling pathways. However, in *HIGD1A*‐silenced COV434 cell lines, the changing trends of those SASP‐associated factors did not exhibit consistency, with MMP1 and TNF‐α increased, IL‐1β, IL‐8, and IL1A decreased, and other factors such as MMP3, CCL20, and IL‐6 not changed significantly. Based on these results, we presumed that SASP may not be the main cause of HIGD1A‐knockdown‐induced cellular senescence, which is consistent with the results reported in pancreatic cancer cell lines.^[^ ^39^ ^]^

Many studies have proved that a large part of apoptotic signaling pathways are mediated by mitochondria.^[^ ^40^ ^]^ The results of this study revealed that although *HIGD1A* deficiency led to aggravation of cellular OS, it did not cause obvious changes in terms of apoptosis.^[^ ^20^ ^]^ It was shown that HIGD1A, one of the important components of mitochondria, serves as a protective factor for DNA‐damage‐induced apoptosis instead of a regulator of mitochondria‐mediated apoptosis.

This study is the first one to systematically investigate the function of *HIGD1A* in GCs and mouse ovaries, revealing that *HIGD1A* deficiency can result in cellular OS damage and serious mitochondrial dysfunction. We then explored corresponding mechanisms responsible for the detrimental impact of *HIGD1A* knockdown. Based on the reported upstream regulatory mechanisms of SOD2,^[^ ^41^, ^42^, ^43^, ^44^ ^]^ we selected several pathway inhibitors, including FOXO1 inhibitor AS1842856, p38 MAPK inhibitor SB203580 and the NF‐κB inhibitor sinomenine, to treat *HIGD1A*‐deficient cells to elucidate how *HIGD1A*‐depletion affects SOD2 upregulation. The results showed that the p38 MAPK inhibitor further increased the level of SOD2, while the FOXO1 inhibitor reduced the HIGD1A level without altering SOD2 expression. Our RNA‐seq data have revealed that *HIGD1A* depletion also led to the inhibition of FOXO1 pathway (including FOXO1, GADD45A, BTG1, and p27), suggesting that HIGD1A and the FOXO1 pathway may form a positive feedback loop that would further affect the expression of each other. Eventually, we found that the NF‐κB inhibitor effectively reduced SOD2 expression in *HIGD1A*‐deficient cells. NF‐κB is one of the key factors regulating inflammatory injury and OS. Activation of STAT3/NF‐κB mediates inflammation and insulin resistance in adipocytokine‐induced hepatocyte injury.^[^ ^45^ ^]^ β2‐AR/FOXO1/p‐NF‐κB pathway is activated during restraint stress in pregnant mice.^[^ ^46^ ^]^ Activation of Cep55/NF‐κB/PI3K/Akt/FOXO1/TNF‐α pathway mediates furosine‐induced toxic effect on Sertoli cells.^[^ ^47^ ^]^ NF‐κB/SOD2 pathway is involved in the anti‐OS protection in the freeze‐tolerant wood frog. In this study, *HIGD1A* deficiency leads to OS in GC cell lines by activating the NF‐κB/SOD2 pathway, which has not been reported in previous studies.

In conclusion, our study showed *HIGD1A* is under the control of different pathways depending on different methods used to establish OS models. As an important mitochondrial protein, HIGD1A plays a key role in regulating mitochondrial functions, OS, GCs behaviors and female fertility. Therefore, developing *HIGD1A*‐based therapies can be a promising direction for antioxidant and female fertility preservation.

## 4 Conclusion

**Figure 9:**
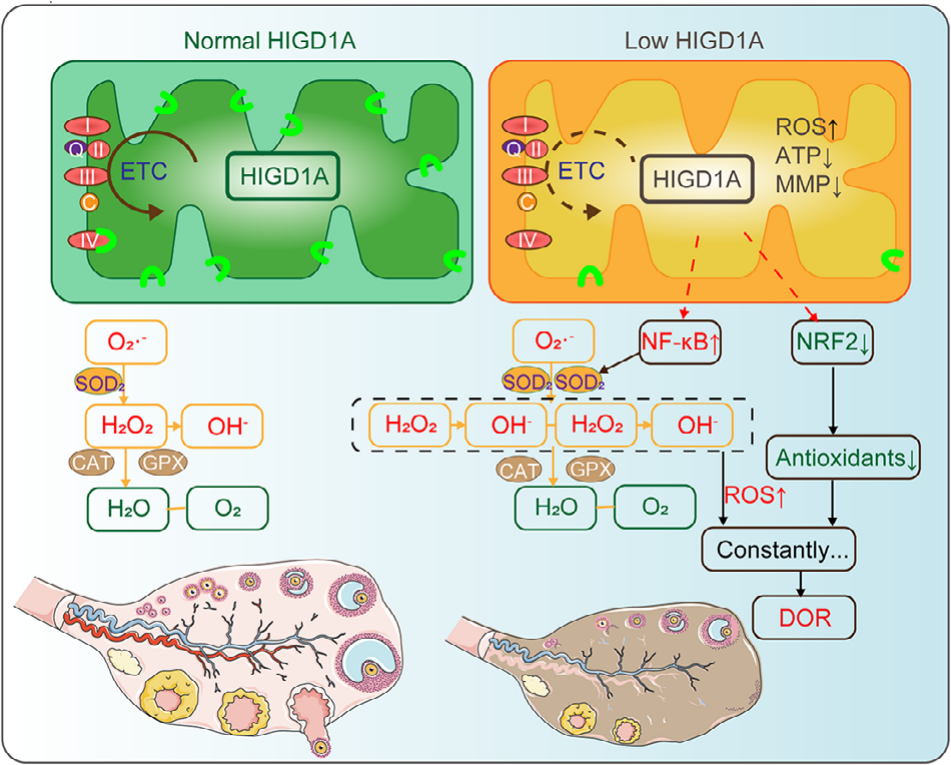
Schematic diagram of the mechanism by which HIGD1A regulates oxidative stress‐mediated ovarian dysfunction. DOR, diminished ovarian reserve; CAT, catalase; SOD, superoxide dismutase; GPX, glutathione peroxidase; ETC, electron transport chain.

These findings elucidated the critical role of *HIGD1A* in regulating GC function and follicular development under OS condition (**Figure** **9**). We revealed that *HIGD1A* is downregulated upon OS exposure, which disturbs proliferation and mitochondrial functions of GC cell lines. Furthermore, we also indicated HIGD1A‐mediated changes in NF‐κB/SOD2 pathway. All these findings provide further insights into the role of HIGD1A in modulating mitochondrial function and OS ‐related cellular response, demonstrating the potential of HIGD1A as a novel therapeutic target to alleviate OS damage and improve female fertility.

## 5 Experimental Section

### Animal Handling and Ethics Approvals

Female C57BL/6J mice (8 weeks old, SPF class) were purchased from Charles River and SiPeiFu, China. After purchase, all mice were maintained under a controlled temperature (26 ± 2 °C) with 12 h light/dark conditions. For in vivo OS models, mice were randomly divided into two groups (3NP, 3NP control, *n* = 20 per group). Mice in the 3NP group received intraperitoneal injections of 3‐itropropionic acid (3NP, Sigma‐Aldrich, USA. 10/20 mg kg^−1^, dissolved in saline, every morning for 14 days), while mice in the control group received intraperitoneal injections of corresponding solvent (normal saline). Vaginal smears were obtained to monitor the estrous cycles of mice.^[^ ^48^ ^]^ Hormone assay and follicle counting were performed using mice at the same phase of the estrous cycle. The body weights of mice were recorded daily. Ovaries were removed and weighed immediately after execution. The ovary coefficient was defined as the ratio of the ovary weight to the body weight of the mouse. For preparation of paraffin sections, the ovaries were immediately fixed in 4% paraformaldehyde, embedded in paraffin, and sectioned into 5‐µm‐thick slices. The paraffin sections were stained with hematoxylin and eosin (H&E) and imaged using a virtual slide scanner VS120 (Olympus, Japan).

Animal experiments were approved by the Institutional Animal Care and Use Committee, Huazhong University of Science and Technology (IACUC Number: S2474) in accordance with the National Research Council's “Guideline for the Care and Use of Laboratory Animals.”

### Ovarian Follicle Count

Serial paraffin sections were obtained from ovarian tissues following the administration of 3‐NP and AAV. Subsequently, every fifth section was subjected to H&E staining to enable the detection and classification of all follicular stages, including primordial, primary, secondary, antral, and atretic, according to established criteria.^[^ ^49^ ^]^ The number of follicles in each ovary was quantified and compared among the experimental groups.

### Sample Collection

The follicular fluid samples were collected from women undergoing oocyte retrieval for IVF/ICSI at the Wuhan Tongji Reproductive Medicine Hospital (Wuhan, China) and Reproductive and Genetic Center of Xiangtan Central Hospital (Xiangtan, China) from March 2019 to December 2020. Participants were required to meet the following requirements: a) age between 20 and 45 years old; b) failed to conceive naturally for at least 1 year; c) primary or secondary infertility; d) received conventional ovulation stimulation treatment. All the samples were divided into two groups: diminished ovarian reserve (DOR) group and normal ovarian reserve (NOR) group. According to the expert consensus of the Chinese Medical Association, patients who meet the following criteria were considered as DOR: a. the AMH level <1.1 ng mL^−1^; b. antral follicle count (AFC) on both sides <5–7. Participants with viral infectious diseases, malignant tumors, immune diseases, chromosomal diseases, mental disorders, and cognitive impairments were excluded. Informed consent was obtained from all participants. This study was approved by the ethics committee of Wuhan Tongji Reproductive Medicine Hospital (Approval. No. 2020 (009); Wuhan, Hubei, China).

### Isolation, Purification, and Identification of Human Granulosa Cells

Human granulosa cells (GCs) were obtained from follicular fluid using a density gradient centrifugation method. Initially, the follicular fluid was subjected to centrifugation at 2500 rpm for 15 min, and the cell pellet was collected. The cells were then resuspended and slowly added onto a 50% Percoll solution (Sigma–Aldrich) followed by centrifugation at 400 g for 30 min. The intermediate layer was collected, and 3 mL of erythrocyte lysis solution was added. Subsequently, the cells were centrifuged at 2500 rpm for 15 min, and the supernatant was discarded. The cell pellet was washed twice with PBS. The purity of the harvested GCs was assessed by performing immunofluorescent staining of the follicle‐stimulating hormone receptor (FSHR). The collected GCs were stored in TRIzol reagent (Invitrogen, USA) at −80 °C until RNA extraction.

### Cell Culture and Treatment

The human granulosa tumor‐derived cell line COV434 was purchased from Wuhan Warner Bio Technology Co., Ltd, China. The human granulosa‐like tumor cell line KGN was purchased from Procell Life Science &Technology Co., Ltd, China. All cells were maintained in Dulbecco's modified Eagle's medium (DMEM, Gibco, USA) with 10% fetal bovine serum (Gibco) and 1% penicillin/streptomycin (Gibco) at 37 °C in an atmosphere comprising 5% CO2, and stored in serum‐free Cell Freezing Medium (Procell) at −80 °C. For in vitro OS models, COV434 and KGN cell lines (70% confluence) were treated with 3NP (Sigma–Aldrich. 0, 2.5, 5, 10, 15, 20, 25, 30 mm in PBS, 24 h) or VK3 (Sigma–Aldrich. 0, 10, 20, 30, 50, 70, 80, 90, 100 µm in DMSO, 24 h) or H_2_O_2_ (SINOPHARM. China. 0, 100, 200, 400, 600, 800, 1000, 1200 µm, 2 h). Transfection of cells with DNA or siRNAs was performed using Lipofectamine 3000 (Thermo Fisher Scientific, USA) following the manufacturer's instructions. For siRNA‐mediated *HIGD1A* knockdown, cells were transfected with 50 nm *HIGD1A*‐directed siRNA as well as its negative controls (RiboBio, China: siN0000001‐4‐10). For *HIGD1A* overexpression, cells were transfected with pcDNA3.1‐*HIGD1A* (Genomeditech Co., Ltd, China) as well as its vectors as negative controls. The siRNA sequences used are provided as follows:

si‐h‐*HIGD1A*_001: 5′‐GGATCAAAACTCATTCGAA‐3′

si‐h‐*HIGD1A*_002: 5′‐GTATGGGCTATTCCATGTA‐3′

si‐h‐*HIGD1A*_003: 5′‐AGCGGGTTTTGCAGCAATT‐3′

### Lentivirus and Adeno‐Associated Virus (AAV) Construction

The lentiviral and AAV vectors used in the study were constructed by Genomeditech. For *HIGD1A* knockdown, the sequence of si‐h‐*HIGD1A*_001 was subcloned into the lentiviral vector PGMLV‐CMV‐MCS‐3×Flag‐Puro (Genomeditech). For AAV‐*Higd1a* constructs, the mouse *Higd1a* cDNA was amplified by PCR, then inserted into the AAV vector GPAAV‐CMV‐EF1‐ZsGreen1‐WPRE (Genomeditech). Lentiviral and AAV particles were prepared at Genomeditech Biotechnology Co., Ltd. To obtain stably transfected cells, COV434 and KGN cell lines were transfected with lentivirus and selected with puromycin. To overexpress *Higd1a* in mouse ovaries, AAV‐*Higd1a* was administered by ovarian local injection.

### Ovarian Local Injection

According to reference,^[^ ^50^ ^]^ *Higd1a* was overexpressed in the ovaries of mice in this study by injecting the overexpressing mice with an epitope AdenoAssociated Virus (AAV) into the bilateral ovarian capsule using an Expression plasmid (GPAAV‐CMV‐Mouse_Higd1a‐EF1‐ZsGreen1‐WPRE) AdenoAssociated Virus (AAV). The adeno‐associated virus was packaged by Jiman Bio at a viral titre of 1E13 VG/mL. Subsequently, 10 µL of viral stock was injected into each ovary while maintaining an equal volume of empty AAV virus as a control. The experiments were performed on 8‐week‐old wild‐type ICR female mice, which were randomly divided into four groups (*n* = 12 per group): NS group (Mice received intraperitoneal injections of physiological saline as a control). 3NP group (Mice received intraperitoneal injections of 3NP (20 mg kg^−1^ day^−1^)). 3NP + AAV‐Control group (3NP was administered intraperitoneally from D14 to D28 after mice received ovarian capsule injection of control AAV). 3NP + AAV‐Higd1a group (3NP was administered intraperitoneally from D14 to D28 after mice received ovarian capsule injection of Higd1a‐overexpressing AAV).

### Cell Viability and Colony Formation Assay

Cell viability was determined with Cell Counting Kit‐8 (CCK‐8, Vazyme, China). Briefly, cells were seeded in 96‐well plates and divided into four groups (*n* = 6). At the indicated time points (every 24 h up to 96 h), 10 µL of CCK‐8 reagent was added to each well, followed by incubation at 37 °C for 1.5 h. The optical absorbance at 450 nm was collected with a Synergy HTX Multi‐Mode Reader (BioTek, USA). Cell proliferation capacity was determined by colony formation assay. Briefly, cells were trypsinized into cell suspension and diluted into the desired seeding concentration. Cells were then seeded in 60 mm dishes at a density of 800 cells per dish and maintained in an incubator at 37 °C. After 14 days of culture, the colonies were fixed and stained with Wright‐Giemsa stain (Servicebio), then the number of colonies in each dish was recorded.

### Flow Cytometry

The cell cycle phase distribution and cellular apoptosis levels were determined using the Cell Cycle Analysis Kit and Annexin V‐PE Apoptosis Detection Kit (Beyotime, China). For cell cycle analysis, cells were fixed with 70% ethanol at 4 °C for 24 h, then incubated with cell cycle staining working solution at 37 °C for 30 min. For cell apoptosis analysis, cells were digested with 0.05% trypsin and incubated with Annexin V‐PE staining solution in the dark for 20 min. Data were collected using NovoCyte Flow Cytometer Systems (Agilent, USA). All the solutions used were prepared according to the manufacturers’ instructions).

### SA‐β‐Gal Staining

SA‐β‐Gal staining for COV434 and KGN cell lines was performed using a Senescence β‐Galactosidase Staining Kit (Beyotime). Briefly, cells were fixed in fixation solution for 15 min and incubated with staining solution at 37 °C overnight. SA‐β‐Gal activity was determined by the ratio of SA‐β‐Gal‐positive cells. All the solutions used were prepared following the manufacturer's instructions.

### Hormone Assays

The mouse blood samples were centrifuged at 1000 g for 10 min to collect serum. The hormone levels were measured using commercialized enzyme‐linked immunosorbent assay (ELISA) kits following the manufacturers’ instructions. The ELISA kits for estradiol (E2), follicle‐stimulating hormone (FSH), and anti‐Müllerian hormone (AMH) were all purchased from Elabscience (China).

### RNA Extraction and Quantitative Real‐Time PCR (qRT‐PCR)

Total RNA was extracted using an RNA‐easy Isolation Reagent (Vazyme, China) and then reversely transcribed to complementary DNA (cDNA) using HiScript II 1st Strand cDNA Synthesis Kit (Vazyme). cDNAs were amplified using Taq Pro Universal SYBR qPCR Master Mix (Vazyme) with a Quantagene q225 real‐time PCR system (Kubo, China). The temperature program was set as follows: 95 °C for 30 s, followed by 40 cycles of 95 °C for 10 s and 60 °C for 30 s. Relative expression of target genes was normalized to β‐actin (ACTB) and fold changes were calculated using the 2^‐ΔΔCt^ method. PCR primers were synthesized by Sangon Biotech (China), as listed in Table S1 (Supporting Information).

### Protein Extraction and Western Blot

Total proteins were extracted using cell lysis buffer (RIPA reagent supplemented with 1×protease inhibitor, 1×phosphatase inhibitor and 100 µm PMSF) and protein concentration was measured by BCA method (Beyotime). 20–50 µg of protein extract was separated by SDS–PAGE and transferred to Polyvinylidene fluoride (PVDF) membrane (Millipore). After blocking, the blots were incubated with indicated primary antibodies and HRP‐conjugated secondary antibodies. The protein bands were detected by an electrochemiluminescence (ECL) reagent kit (EpiZyme, China) and imaged with Bio‐Rad Gel Imaging Systems (Bio‐Rad, USA). Antibodies used are indicated in Table S2 (Supporting Information).

### Immunofluorescence (IF) Staining

For cultured cells, cells were fixed with 4% paraformaldehyde and permeabilized with 0.5% Triton X‐100. For mouse ovarian tissue, the paraffin sections were first dewaxed and rehydrated, then boiled with antigen retrieval buffer (Servicebio) for 10 min. The cells and sections were then blocked with 3% goat serum and incubated with indicated primary antibodies (4 °C overnight) and secondary antibodies(RT, 1 h). For nuclear staining, the cells and sections were incubated with fluorescent dye DAPI staining reagent (Servicebio) for 5 min. Fluorescence images were captured using a Zeiss Axio Observer 5 fluorescence microscope (Carl Zeiss). The antibodies used are indicated in Table S2 (Supporting Information).

### Measurement of ATP Levels

The changes in ATP levels were measured using an ATP Assay Kit (Beyotime, China). Briefly, cells were lysed with ATP lysis buffer then centrifuged at 12 000 g, 4 °C for 5 min. The supernatant was collected for subsequent measurement. To remove background ATP, working solution was added to opaque 96‐well plate, incubated for 5 min and removed. Then the reference standard and samples were added to the detection well, and the relative light unit (RLU) values were immediately collected with a Synergy HTX Multi‐Mode Reader (BioTek). All the solutions used in this assay were prepared following the manufacturers’ instructions.

### Extraction and Quantification of mtDNA

mtDNA was extracted and purified using the TIANamp Genomic DNA kit (TIANGEN, China) following manufacturer's instructions. The copy numbers of mtDNA were analyzed via amplification using ND1 and β‐globin primers and RT‐qPCR. The primers used are listed in Table S1 (Supporting Information).

### Intracellular ROS Quantification

A reactive oxygen species assay kit (Beyotime) was used to determine the intracellular ROS levels. The cells were rinsed and incubated with the fluorescent probe DCFH‐DA in the dark at 37 °C for 20 min, followed by incubat ion with Hoechst 33342 for nuclear staining. Fluorescent images were captured with a Zeiss LSM 900 Confocal Laser Scanning Microscope (Carl Zeiss, Germany). Quantification of intracellular ROS was performed by analyzing the fluorescent intensity.

### Detection of Cellular Mitochondrial Distribution

To visualize cellular mitochondrial distribution, cells were incubated with Mito‐Tracker Red CMXRos (200 nm, 30 min) together with Hoechst 33342 according to the manufacturer's protocol. After incubation, the images were captured with a Zeiss LSM 900 Confocal Laser Scanning Microscope (Carl Zeiss).

### Measurement of Mitochondrial Membrane Potential (MMP)

The changes in cellular MMP were assessed by a commercialized JC‐10 kit (Solarbio). Briefly, cells were incubated with JC‐10 working solution in the dark at 37 °C for 25 min and washed 2 times with 1× JC‐10 staining buffer. Then cells were incubated with Hoechst 33342 for nuclear staining. The fluorescent images of JC‐10 polymer(red)/monomer(green) were captured at emission wavelengths of 590/530 nm upon excitation wavelengths of 525/490 nm, respectively. The cellular MMP was represented as the polymer/monomer ratio. All the solutions used were prepared following the manufacturers’ instructions.

### Evaluation of Oxidative Stress (OS) Levels

The OS levels of GCs and mouse ovaries were determined by evaluating total antioxidant capacity(T‐AOC), enzyme activities of catalase (CAT), superoxide dismutase (SOD) and myeloperoxidase (MPO), lipid peroxidation (LPO) levels and malondialdehyde (MDA) levels. All the evaluations were performed using commercialized kits (Nanjing Jiancheng Bioengineering Institute, China) according to the manufacturers’ instructions.

### RNA Sequencing and Data Analysis

Total RNA was extracted using Trizol reagent (Invitrogen) as described by the manufacturer. The quality and concentration of RNA samples were analyzed by NanoDrop 2000 Spectrophotometer (Thermo Fisher, USA). Library construction and paired‐end RNA‐seq assays were performed on an Illumina NovaSeq 6000 platform by Annaroad Gene Technology Co., Ltd (China). Raw sequencing reads were aligned to the USCS hg38.0 *Homo sapiens* reference genome using HISAT2 aligner v2.1.0 with default settings. Gene counts were computed with HTSeq v0.6.0. Differentially expressed genes (DEGs) was determined by DESeq2 v1.24.0. Significant DEGs were defined with the criteria |log_2_(fold change) | >1 and *p* value <0.05. KEGG enrichment analysis and gene set enrichment analysis (GSEA) were performed with clusterProfiler R package v3.18.1.

### Statistical Analysis

Statistical analyses and visualization were performed using GraphPad Prism (version 8.0c; GraphPad Software, Inc., USA). Adobe Illustrator was used to compile and generate all figures in their final form. For dose‐response curves, where a nonlinear regression model fitting a variable slope for (log[x] vs normalized viability response) was used to determine IC50 and significance. The quantification data were presented as mean ± standard error and statistical significance of equally distributed data was tested by Student's *t*‐test or single‐factor analysis of variance (one‐way ANOVA). Each experiment was independently repeated at least three times (N), with at least four technical replicates (*n* ≥ 4) per condition to ensure data reliability. Differences with *p* values < 0.05 were considered statistically significant. For all statistical analyses, asterisks representing *p* values are indicated in each figure legend, with ns indicating “not significant” (*p* > 0.05).

## Conflict of Interest

The authors declare no conflict of interest.

## Author Contributions

H.L., S.C., and H.M. contributed equally to this study. Initial drafting of the manuscript (HYL), revision of the manuscript (SYC, WPX), data collection (HYL, SYC, HBM, TLC, XFW, TYT, TYD, YZ), data analysis (HYL, HBM, SYC), figure design (HYL, HBM, LQZ), initial study design (HBL, WPX), experimental oversight and guidance (ZL, HBL, LQZ, WPX).

## Supporting information



Supporting Information

## Data Availability

The data that support the findings of this study are available from the corresponding author upon reasonable request.
