# Supplementary material for: HIGD1A Alleviates Oxidative Stress Related Ovarian Hypofunction by Enhancing Granulosa Cell Functions via NF‐κB/SOD2 Signaling Pathway
Source: Adv Sci (Weinh). 2025 Aug 13;12(34):e03828. doi: 10.1002/advs.202503828 (PMC12442643; doi:10.1002/advs.202503828)
Supplement: Supplementary file 1 — Supporting Information [file ADVS-12-e03828-s001.docx]

**Supplementary file:**


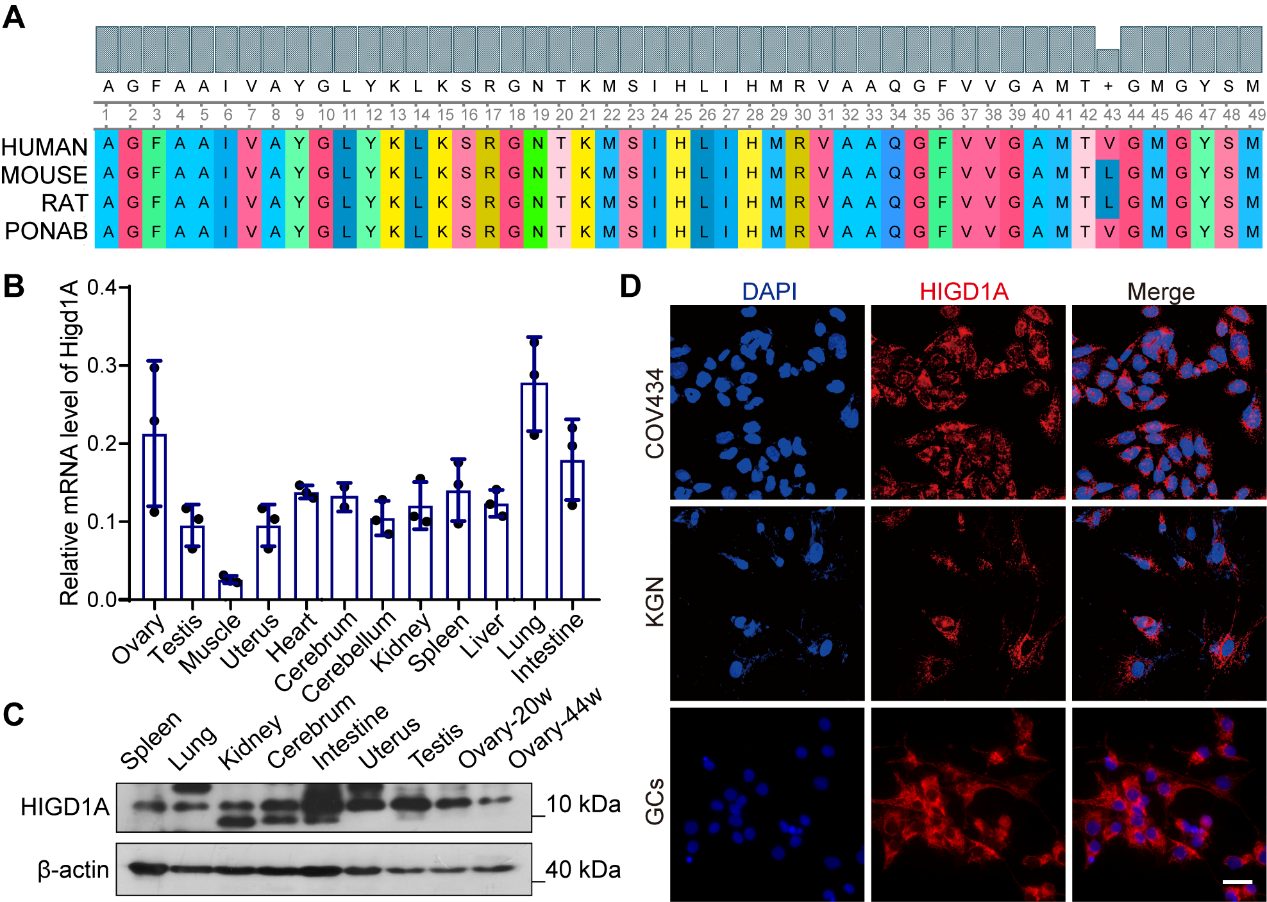


**Figure S1. Expression pattern of HIGD1A in different organs of ICR mice and granulosa cells.**

(A) Multiple sequence alignment of human, mouse, rat and ponab HIGD1A protein sequences. (B, C) RT-qPCR and western blot analysis of Higd1a mRNA and protein levels in different mouse organs. β-actin served as loading control(n ≥ 3). (D) HIGD1A immunofluorescent staining showing the subcellular localization pattern of HIGD1A in COV434, KGN and human granulosa cells (GCs) (n ≥ 3). The experiment was repeated three times and the results were expressed as mean ± standard error and analyzed by t-test.


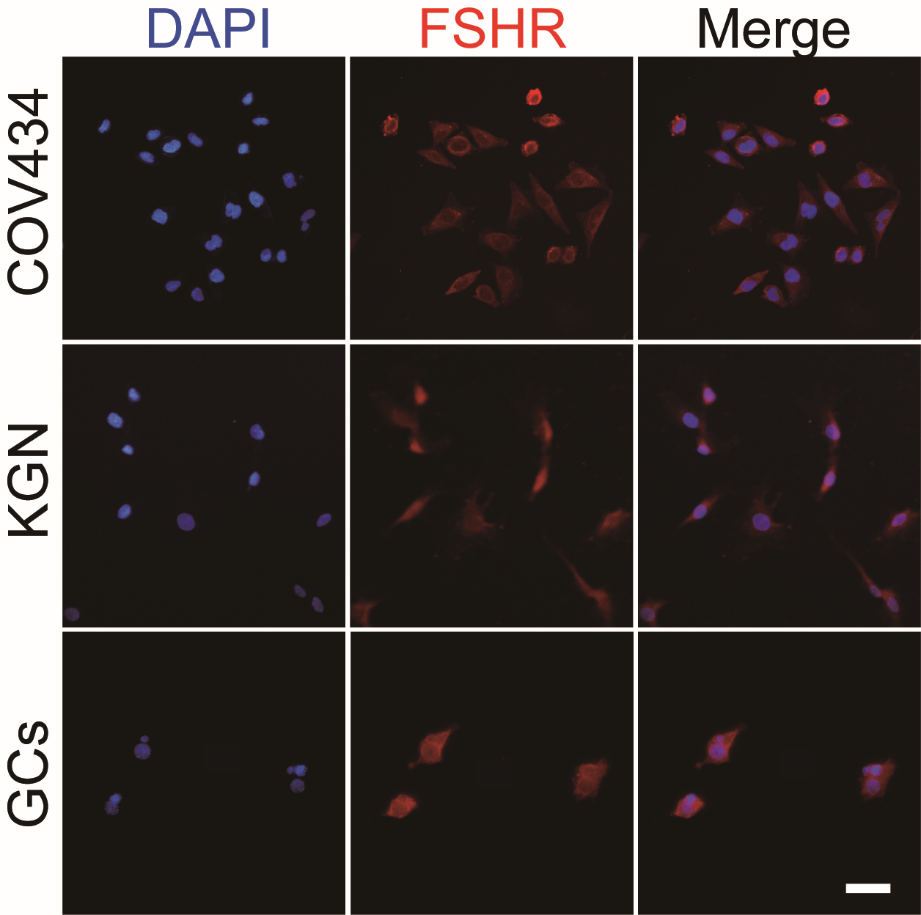


**Figure S2. COV434, KGN and primary granulosa cells (GCs) identified by FSHR immunofluorescent staining.** Scale bar: 40μm.


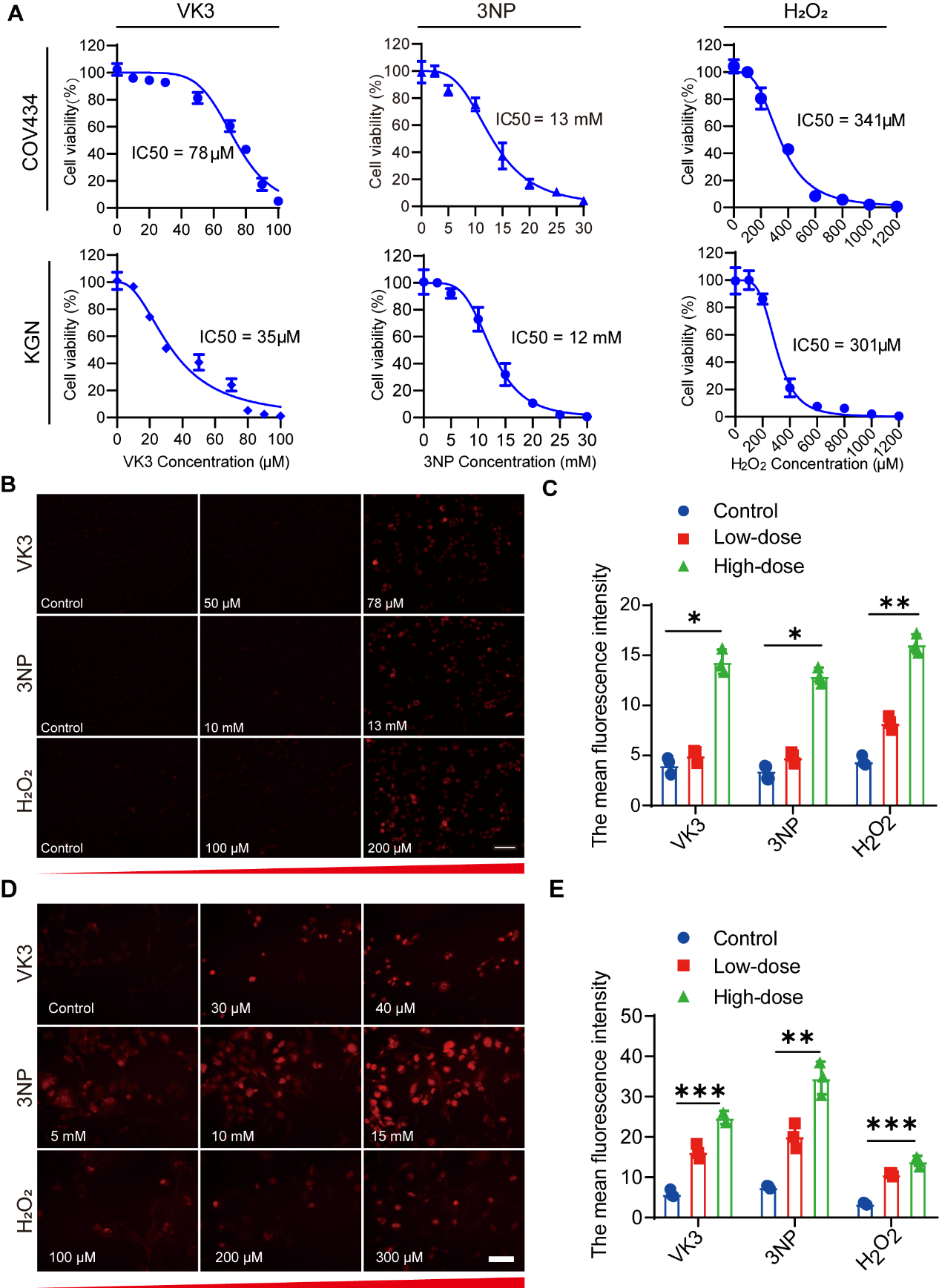


**Figure S3. in vitro OS models of COV434 and KGN cells induced by VK3, 3NP and H_2_O_2_.** (A) IC50 concentrations of indicated OS inducers in COV434 and KGN cells were obtained by CCK-8 assay (n=6). (B, C) ROS generation upon treatment of different drugs at indicated concentrations in COV434 cells was visualized by DCFH-DA probe. (D, E) ROS generation upon treatment of different drugs at indicated concentrations in KGN cells was visualized by DCFH-DA probe. The experiment was repeated three times and the results were expressed as mean ± standard error and analyzed by t-test (n ≥ 3). *, P < 0.05, **, P < 0.01, ***, P < 0.001.

**
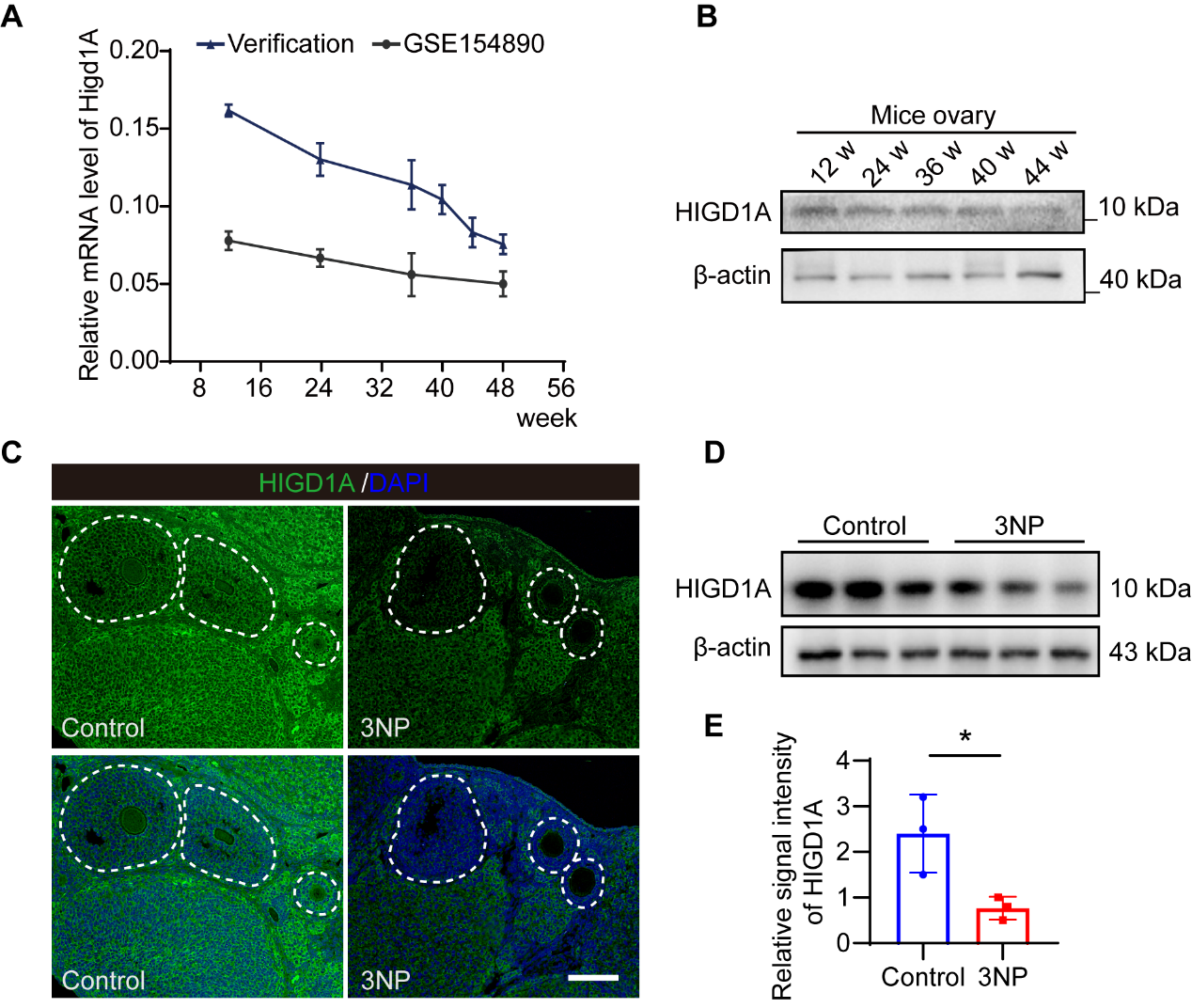
**

**Figure S4. Higd1a is downregulated in GCs in aged mice and 3NP induced OS models.**

(A) *Higd1a* mRNA levels of ovaries from mice of different ages were acquired from GEO database. (B) Representative western blot analysis of HIGD1A protein levels of ovaries from mice of different ages. β-actin served as loading control. (C) HIGD1A immunofluorescent staining in mouse ovaries with or without 3NP treatment. (D-E) HIGD1A protein levels of mouse ovaries were determined by western blot analysis. β-actin served as loading control. The experiment was repeated three times and the results were expressed as mean ± standard error and analyzed by t-test (n ≥ 3). *, *P* < 0.05.


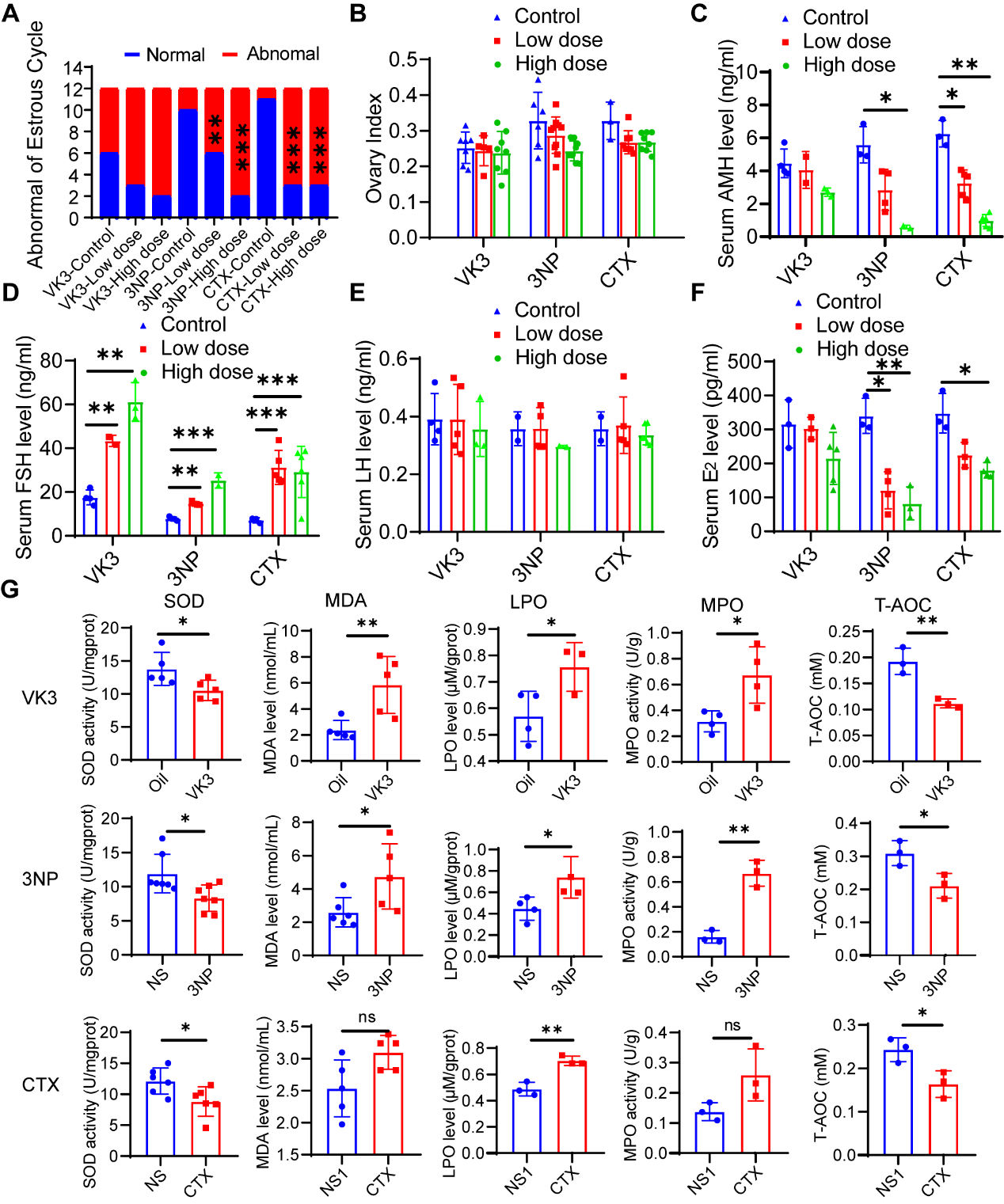


**Figure S5. *in vivo OS models of ICR mice induced by VK3, 3NP and CTX.***

(A) Estrous cycle disorders induced by treatment of different drugs in ICR mice. (B) Changes in ovary index upon treatment of different drugs were measured. (C-F) Levels of AMH, FSH, LH and E2 in serum samples were determined by ELISA. (G) Levels of OS markers changed after VK3/3NP/CTX treatment. The experiment was repeated three times and the results were expressed as mean ± standard error and analyzed by t-test (n ≥ 3). *, P < 0.05, **, P < 0.01, ***, P < 0.001.


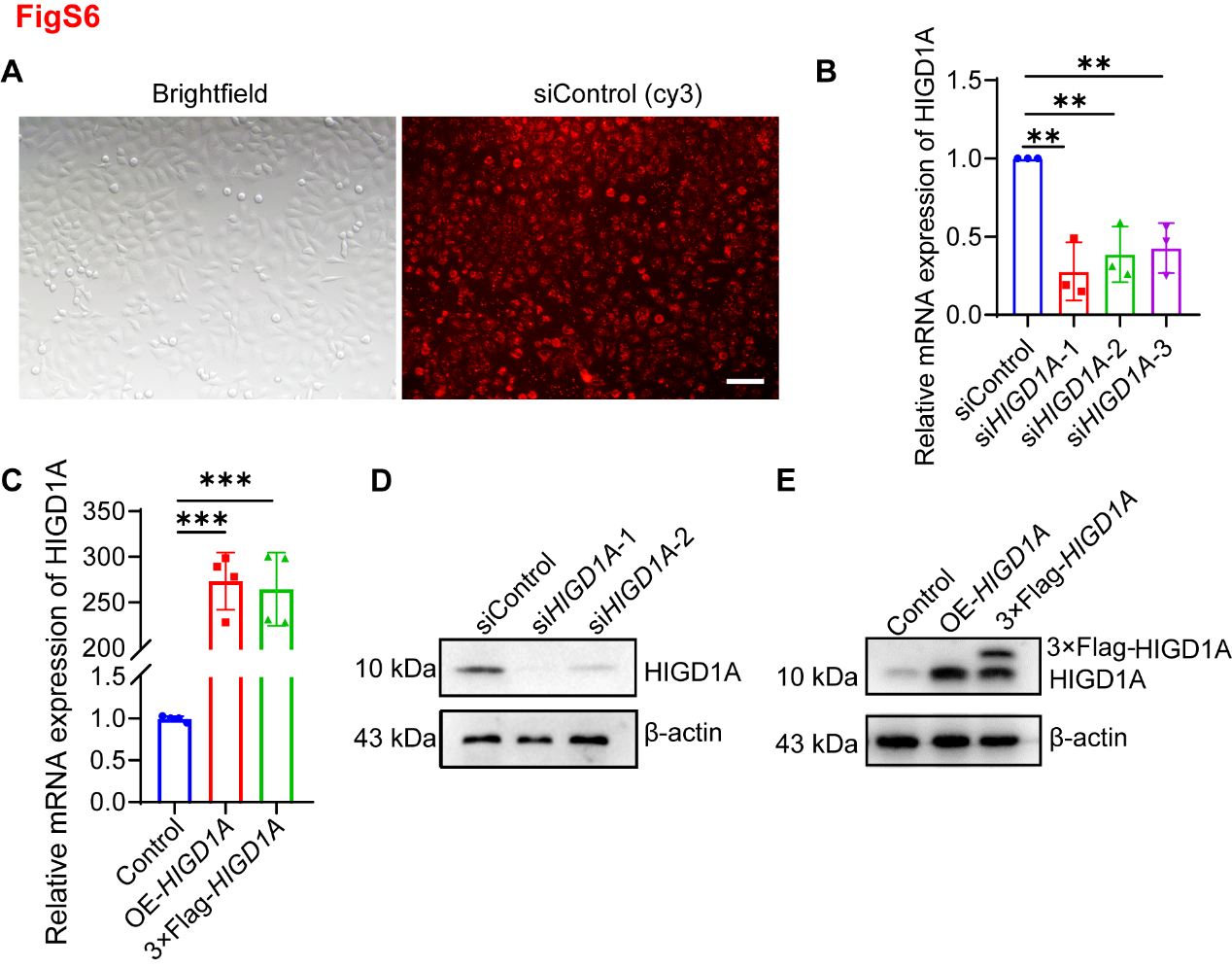


**Figure S6. Knockdown or overexpression efficiency of *HIGD1A*.**

(A) Representative brightfield and fluorescent images of COV434 cells after transfection of cy3-labelled siRNA. (B, C) *HIGD1A* mRNA levels after siRNA-mediated knockdown or overexpression were determined by RT-qPCR. (D, E) HIGD1A protein levels after siRNA-mediated knockdown or overexpression were determined by western blot analysis. β-actin served as loading control. The experiment was repeated three times and the results were expressed as mean ± standard error and analyzed by t-test (n ≥ 3). **, P < 0.01, ***, P < 0.001.


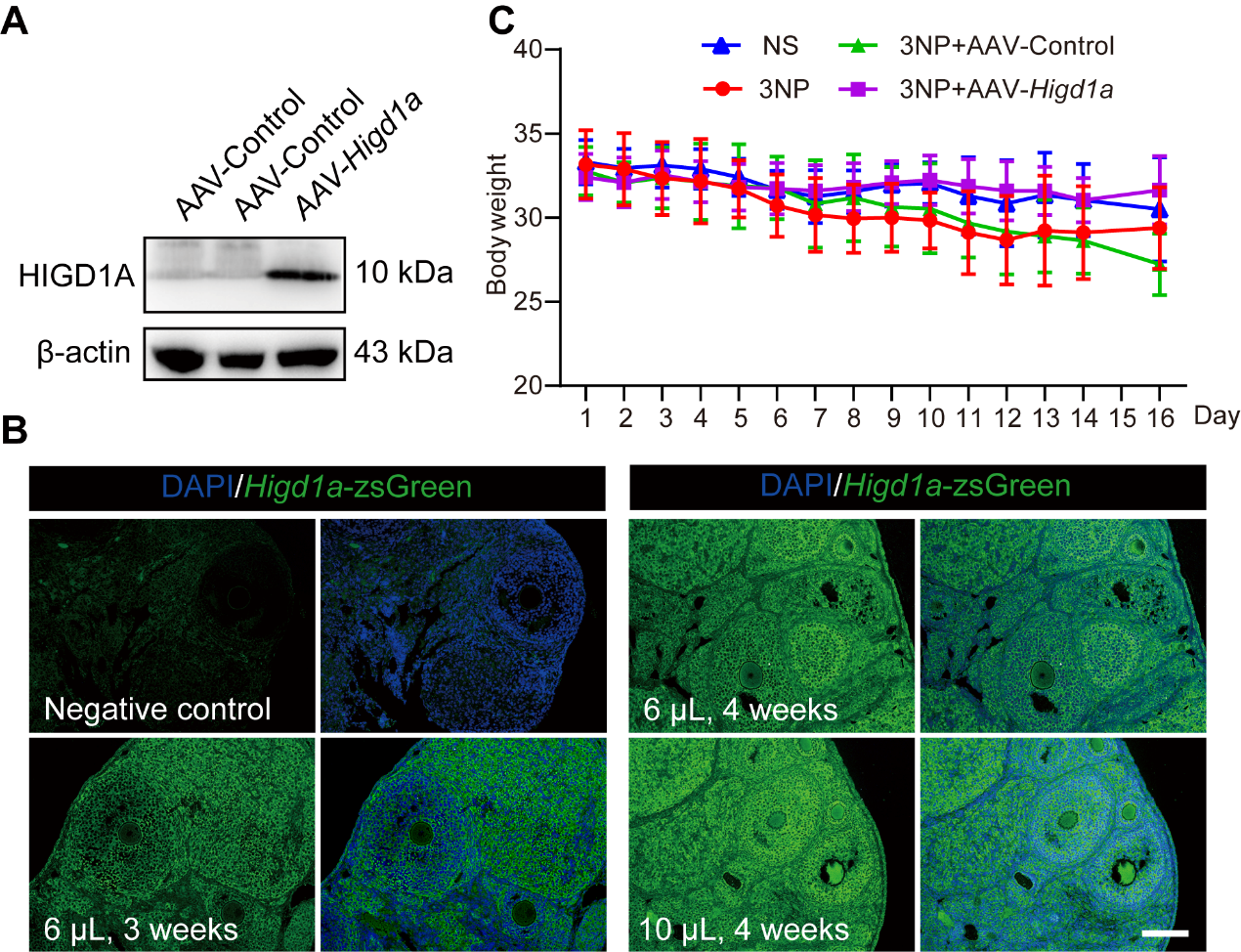


**Figure S7. AAV-mediated *Higd1a* overexpression in mouse ovaries.**

(A) AAV-mediated *Higd1a* overexpression efficiency was verified in HEK293T cells (n=3). (B) GFP fluorescent images showing transfection efficiency of AAV ovarian local injection (n=3). (C) Body weights of mice received 3NP and AAV administration were recorded (n=12 per group).


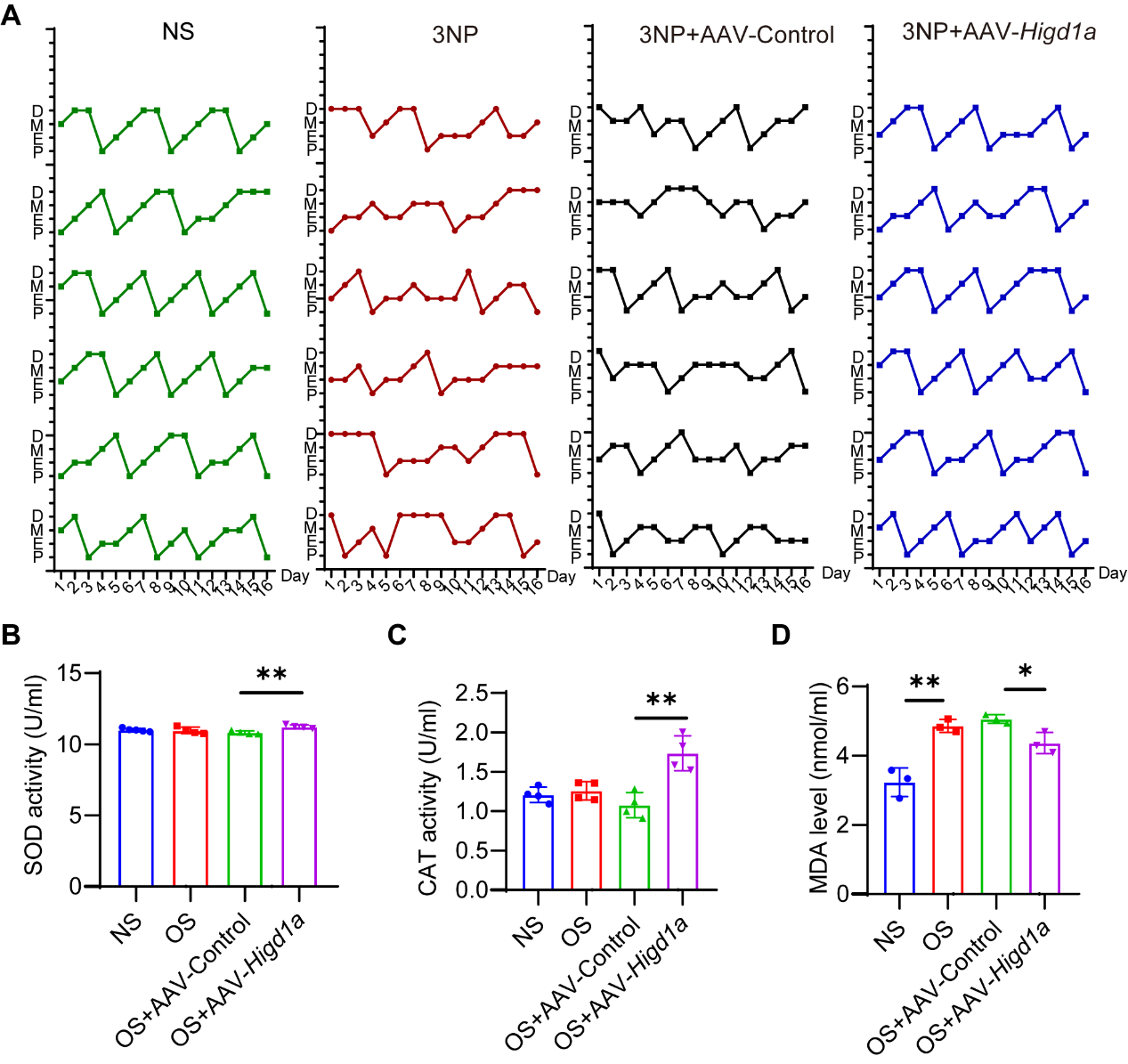


**Figure S8. *Higd1a* overexpression alleviates estrous cycle disorder and OS in mouse ovaries.**

(A) Estrous cycles of mice during administration of 3NP and AAV were recorded (n=12 per group). (B-D) The impact of AAV-mediated *Higd1a* overexpression on SOD and CAT activities and MDA levels of mouse ovaries were measured. The experiment was repeated three times and the results were expressed as mean ± standard error and analyzed by t-test (n ≥ 3). *, *P* < 0.05, **, *P* < 0.01.


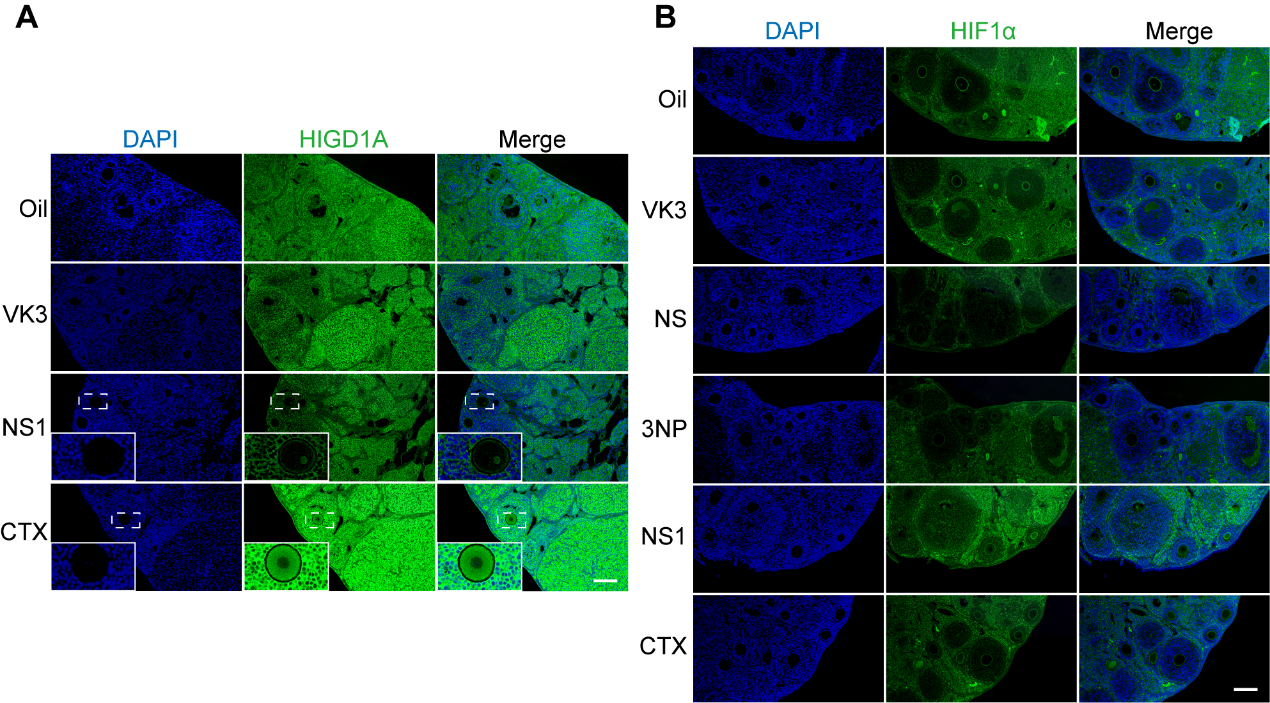


**Figure S9. Expression and localization of HIGD1A and HIF-1α in mouse ovaries upon different treatment.**

(A) Immunofluorescent staining of HIGD1A in mouse ovaries after VK3/CTX treatment. (B) Immunofluorescent staining of HIF-1α in mouse ovaries after 3NP/VK3/CTX treatment. The experiment was repeated three times. Scale bar, 100μm.

### Supplementary tables

#### Supplementary Table 1 The primer sequences used in this study

| **Primer** | **Forward Sequence (5'->3')** | **Reverse Sequence (5'->3')** |
| --- | --- | --- |
| *HS-ACTB* | Sangon Biotech (Order NO. B661102) | |
| *MM-ACTB* | Sangon Biotech (Order NO. B661302) | |
| *CASP3-h* | 5′-CATGGAAGCGAATCAATGGACT-3' | 5′-CTGTACCAGACCGAGATGTCA-3' |
| *CASP9-h* | 5′-CTCAGACCAGAGATTCGCAAAC-3' | 5′-GCATTTCCCCTCAAACTCTCAA-3' |
| *Cat-m* | 5′-AGCGACCAGATGAAGCAGTG-3' | 5′-TCCGCTCTCTGTCAAAGTGTG-3' |
| *CCL20-h* | 5′-AGAGTTTGCTCCTGGCTGCTTTG-3' | GGATGAAGAATACGGTCTGTG-3' |
| *CDK1-h* | 5′-GATTCTATCCCTCCTGGTC-3' | 5′-AATATGGTGCCTATACTCC-3' |
| *CDKN1A-h* | 5′-CCTGTCACTGTCTTGTACCCT-3' | 5′-GCGTTTGGAGTGGTAGAAATC-3' |
| *CDKN2A-h* | 5′-CGGTCGGAGGCCGATCCAG-3' | 5′-GCGCCGTGGAGCAGCAGCAGCT-3' |
| *CDKN2B-h* | 5′-GGATCCCAACGGAGTCAACC-3' | 5′-GCAGGTACCCTGCAACGTC-3' |
| *CXCL1-h* | 5′-GCAGGGAATTCACCCCAAGAAC-3' | 5′-TTCTTAACTATGGGGGATGCAGGA-3' |
| *Cyclin D1-h* | 5′-ACTTCAAATGTGTGCAGAAGG-3' | 5′-GTAGTAGGACAGGAAGTTGTT-3' |
| *Cyclin E-h* | 5′-CTGGATGTTGACTGCCTTGA-3' | 5′-CCGCTGCTCTGCTTCTTAC-3' |
| *CYP11A1-h* | 5′-CAGTCATCCTAGCAGTCCCC-3' | 5′-GGGGATCTCATTGAAGGGGC-3' |
| *CYP17A1-h* | 5′-GCTGCTTACCCTAGCTTATTTGT-3' | 5′-ACCGAATAGATGGGGCCATATTT-3' |
| *CYP19A1-h* | 5′-ACTACAACCGGGTATATGGAGAA-3' | 5′-TCGAGAGCTGTAATGATTGTGC-3' |
| *DHFR-h* | 5′-ATGGTCTGGATAGTTGGTGGC-3' | 5′-GTCTTGCATGATCCTTGTCAC-3' |
| *DRP1-h* | 5′-CTGCCTCAAATCGTCGTAGTG-3' | 5′-GAGGTCTCCGGGTGACAATTC-3' |
| *Drp1-m* | 5′-TTACGGTTCCCTAAACTTCACG-3' | 5′-GTCACGGGCAACCTTTTACGA-3' |
| *FIS1-h* | 5′-GATGACATCCGTAAAGGCATCG-3' | 5′-AGAAGACGTAATCCCGCTGTT-3′ |
| *Fis1-m* | 5′-TGTCCAAGAGCACGCAATTTG-3' | 5′-CCTCGCACATACTTTAGAGCCTT-3' |
| *Gpx-m* | 5′-AGTCCACCGTGTATGCCTTCT-3' | 5′-GAGACGCGACATTCTCAATGA-3′ |
| *HIGD1A-h* | 5′-GTCTTGTTGGAGGAGCTTGC-3' | 5′-ATCAAGCCTGCAAGAAAGGA-3' |
| *Higd1a-m* | 5′-TAAGGAGACACCGTTTGTCCC-3' | 5′-TGATACATGGAGTAGCCCATACC-3' |
| *HS-ACTB* | Sangon Biotech (Order NO. B661102) | |
| *HSD17B1-h* | 5′-ATGACGTTTATTGCGCCAGC-3' | 5′-GGTGTTGACTCACTGGACCC-3' |
| *IL1A-h* | 5′-AGTGCTGCTGAAGGAGATGCCTGA-3' | 5′-CCCCTGCCAAGCACACCCAGTA-3' |
| *IL-1β-h* | 5′-TTTGAGTCTGCCCAGTTCCC-3' | 5′-TCAGTTATATCCTGGCCGCC-3' |
| *IL6-h* | 5′-CCAGGAGCCCAGCTATGAAC-3' | 5′-CCCAGGGAGAAGGCAACTG-3' |
| *IL8-h* | 5′-GAGTGGACCACACTGCGCCA-3' | 5′-TCCACAACCCTCTGCACCCAGT-3' |
| *MFN1-h* | 5′-TGGCTAAGAAGGCGATTACTGC-3' | 5′-TCTCCGAGATAGCACCTCACC-3′ |
| *Mfn1-m* | 5′-ATGGCAGAAACGGTATCTCCA-3' | 5′-CTCGGATGCTATTCGATCAAGTT-3' |
| *MFN2-h* | 5′-CTCTCGATGCAACTCTATCGTC-3' | 5′-TCCTGTACGTGTCTTCAAGGAA-3' |
| *Mfn2-m* | 5′-ACCCCGTTACCACAGAAGAAC-3' | 5′-AAAGCCACTTTCATGTGCCTC-3' |
| *MMP1-h* | 5′-CCTGCAGTTGAACCAGCTAT-3' | 5′-CATGACTTTCCTGGAATTGG-3' |
| *MMP2-h* | 5′-TACAGGATCATTGGCTACACACC-3' | 5′-GGTCACATCGCTCCAGACT-3' |
| *MMP3-h* | 5′-TGTGAGTGAGTGATAGAGTGGG-3' | 5′-ATGGACAAAGGATACAACAGGGA-3' |
| *MMP9-h* | 5′-AGACCTGGGCAGATTCCAAAC-3' | 5′-CGGCAAGTCTTCCGAGTAGT-3' |
| *NRF2-h-F* | 5′-TCCAGTCAGAAACCAGTGGAT-3' | 5′-GAATGTCTGCGCCAAAAGCTG-3' |
| *NRF2-m* | 5′-TCTTGGAGTAAGTCGAGAAGTGT-3' | 5′-GTTGAAACTGAGCGAAAAAGGC-3' |
| *OPA1-h* | 5′-AGCCTCGCAGGAATTTTTGG-3' | 5′-TGTCCTTAATTGGGGTCGTTG-3' |
| *Opa1-m* | 5′-TGGAAAATGGTTCGAGAGTCAG-3' | 5′-AGCCGATCCTAGTATGAGATAGC-3' |
| *p53-h* | 5′-GAGGTTGGCTCTGACTGTACC-3' | 5′-TCCGTCCCAGTAGATTACCAC-3' |
| *p16-h* | 5′-GGGTTTTCGTGGTTCACATCC-3' | 5′-CTAGACGCTGGCTCCTCAGTA-3' |
| *p19^ARF^-h* | 5′-CCTCAACCGCTTCGGCAAGA-3' | 5′-AGGATGTCCACGAGGTCCTGA-3' |
| *p21-h* | 5′-CGATGGAACTTCGACTTTGTCA-3' | 5′-GCACAAGGGTACAAGACAGTG-3' |
| *P27^KIP1^-h* | 5′-AACGTGCGAGTGTCTAACGG-3' | 5′-TGCAGGTCGCTTCCTTATTCC-3' |
| *PGC-1α-h* | 5′-GCTTTCTGGGTGGACTCAAGT-3' | 5′-GAGGGCAATCCGTCTTCATCC-3' |
| *Pgc-1α-m* | 5′-TATGGAGTGACATAGAGTGTGCT-3' | 5′-GTCGCTACACCACTTCAATCC-3' |
| *RPS14-h* | 5′-CTGCGAGTGCTGTCAGAGG-3' | 5′-TCACCGCCCTACACATCAAACT-3' |
| *SIRT1-h* | 5′-TAGCCTTGTCAGATAAGGAAGGA-3' | 5′-ACAGCTTCACAGTCAACTTTGT-3' |
| *Sirt1-m* | 5′-TGACAGAACGTCACACGCC-3' | 5′-AACAATCTGCCACAGCGTCA-3' |
| *SIRT2-h* | 5′-TGCGGAACTTATTCTCCCAGA-3' | 5′-GAGAGCGAAAGTCGGGGAT-3' |
| *Sirt2-m* | 5′-GCCTGGGTTCCCAAAAGGAG-3' | 5′-GAGCGGAAGTCAGGGATACC-3' |
| *SIRT3-h* | 5′-GACATTCGGGCTGACGTGAT-3' | 5′-ACCACATGCAGCAAGAACCTC-3' |
| *Sirt3-m-F* | 5′-ATCCCGGACTTCAGATCCCC-3' | 5′-CAACATGAAAAAGGGCTTGGG-3' |
| *SOD1-h-F* | 5′-GGTGGGCCAAAGGATGAAGAG-3' | 5′-CCACAAGCCAAACGACTTCC-3' |
| *Sod1-m-F* | 5′-AACCAGTTGTGTTGTCAGGAC-3' | 5′-CCACCATGTTTCTTAGAGTGAGG-3' |
| *SOD2-h-F-* | 5′-TTTCAATAAGGAACGGGGACAC-3' | 5′-GTGCTCCCACACATCAATCC-3' |
| *Sod2-m-F* | 5′-CAGACCTGCCTTACGACTATGG-3' | 5′-CTCGGTGGCGTTGAGATTGTT-3' |
| *StAR-h-F* | 5′-GGGATGAGGCTCTTGGATT-3' | 5′-CCCATATCAGCCACTAGCAT-3' |
| *TK1-h-F* | 5′-ACAAGTGCCTGGTGATCAAGTA-3' | 5′-AGTGCAGCCACAATTACGG-3' |
| *TNFα-h-F* | 5′-GAGGCCAAGCCCTGGTATG-3' | 5′-CGGGCCGATTGATCTCAGC-3' |

The nomination marking "h" indicates the specific species as "Homo sapiens (human)", and the marking "m" indicates the specific species as "Mus musculus (mouse)".

#### Supplementary Table 2 The antibodies used in this study

| **Antibody** | **Source (Identifier)** |
| --- | --- |
| Acetylated-Lysine Antibody | Cell Signaling Technology (9441s) |
| AIF Polyclonal Antibody | Proteintech (17984-1-AP) |
| Alpha Tubulin Monoclonal Antibody | Proteintech (66031-1-Ig) |
| ATP5A1 Monoclonal Antibody | Proteintech (66037-1-Ig) |
| Bcl-2 (D17C4) Rabbit mAb | Cell Signaling Technology (3498t) |
| Beta Actin Monoclonal antibody | Proteintech (66009-1-Ig) |
| BTG1 Polyclonal Antibody | Proteintech (14879-1-AP) |
| C1QBP Polyclonal Antibody | Proteintech (24474-1-AP) |
| Catalase Polyclonal antibody | Proteintech (21260-1-AP) |
| Clusterin Polyclonal Antibody | Proteintech (12289-1-AP) |
| COXIV mouse monoclonal antibody | Proteintech (60251-1-Ig) |
| DRP1 (C-Terminal) Polyclonal Antibody | Proteintech (12957-1-AP) |
| DRP1 (D6C7) Rabbit mAb | Cell Signaling Technology (8570) |
| DYKDDDDK Tag Antibody (Binds to same epitope as Sigma’s Anti-FLAG® M2 Antibody) #2368 | Cell Signaling Technology (2368t) |
| DYKDDDDK Tag Polyclonal Antibody (Binds To FLAG® Tag Epitope) | Proteintech (20543-1-AP) |
| FIS1 Monoclonal Antibody | Proteintech (66635-1-Ig) |
| FOXO1 Polyclonal Antibody | Proteintech (18592-1-AP) |
| FSHR Polyclonal antibody | Proteintech (22665-1-AP) |
| GADD45A Polyclonal Antibody | Proteintech (13747-1-AP) |
| HIGD1A Polyclonal Antibody | Santa Cruz (Sc-99441) |
| HIGD1A Polyclonal Antibody | Huabio (Customized) |
| IPKine IgG light chain antibody, HRP labeling | Abbkine |
| IκBα (44D4) Rabbit mAb | Cell Signaling Technology (4812) |
| KI67 Polyclonal Antibody | Proteintech (27309-1-AP) |
| MFN1 Monoclonal antibody | Proteintech (66776-1-Ig) |
| Mouse IgG Mouse Polyclonal | Proteintech (B900620) |
| NF-κB P65 Monoclonal Antibody | Proteintech (66535-1-Ig) |
| NRF1 Monoclonal Antibody | Proteintech (66832-1-Ig) |
| OPA1 Monoclonal Antibody | Proteintech (66583-1-Ig) |
| P27KIP1 Polyclonal Antibody | Proteintech (25614-1-AP) |
| PGC1a Monoclonal Antibody | Proteintech (66369-1-Ig) |
| Phospho-Histone H2A.X (Ser139) Antibody | Cell Signaling Technology (2577s) |
| Phospho-IκBα (Ser32) (14D4) Rabbit mAb | Cell Signaling Technology (2859) |
| Protein G Magnetic Beads | Cell Signaling Technology (70024s) |
| SIRT1 Monoclonal Antibody | Proteintech (60303-1-Ig) |
| SLC25A3 Polyclonal Antibody | Proteintech (10420-1-AP) |
| SLC25A3 Antibody (F-1) | Santa Cruz (Sc-376742) |
| SOD1 Polyclonal Antibody | Proteintech (10269-1-AP) |
| SOD2 Monoclonal Antibody | Proteintech (66474-1-Ig) |
| Anti-FLAG M2 Magnetic Beads | Millipore (M8823) |
| Goat Anti-Rabbit IgG H&L (Alexa Fluor® 647) | Abcam (Ab150083) |
